# High transmission efficiency of the simian malaria vectors and population expansion of their parasites *Plasmodium cynomolgi* and *Plasmodium inui*

**DOI:** 10.1371/journal.pntd.0011438

**Published:** 2023-06-29

**Authors:** Nantha Kumar Jeyaprakasam, Van Lun Low, Sandthya Pramasivan, Jonathan Wee Kent Liew, Wan-Yusoff Wan-Sulaiman, Indra Vythilingam

**Affiliations:** 1 Faculty of Medicine, Department of Parasitology, Universiti Malaya, Kuala Lumpur, Malaysia; 2 Faculty of Health Sciences, Biomedical Science Program, Center for Toxicology and Health Risk Studies, Universiti Kebangsaan Malaysia, Kuala Lumpur, Malaysia; 3 Tropical Infectious Diseases Research and Education Centre (TIDREC), Universiti Malaya, Kuala Lumpur, Malaysia; 4 Environmental Health Institute, National Environment Agency, Singapore, Singapore; Kenya Agricultural and Livestock Research Organization, KENYA

## Abstract

**Background:**

The elimination of malaria in Southeast Asia has become more challenging as a result of rising knowlesi malaria cases. In addition, naturally occurring human infections with other zoonotic simian malaria caused by *Plasmodium cynomolgi* and *Plasmodium inui* adds another level of complexity in malaria elimination in this region. Unfortunately, data on vectors which are responsible for transmitting this zoonotic disease is very limited.

**Methodology/Principal findings:**

We conducted longitudinal studies to investigate the entomological parameters of the simian malaria vectors and to examine the genetic diversity and evolutionary pattern of their simian *Plasmodium*. All the captured *Anopheles* mosquitoes were dissected to examine for the presence of oocysts, sporozoites and to determine the parous rate. Our study revealed that the *Anopheles* Leucosphyrus Group mosquitoes are highly potential competent vectors, as evidenced by their high rate of parity, survival and sporozoite infections in these mosquitoes. Thus, these mosquitoes represent a risk of human infection with zoonotic simian malaria in this region. Haplotype analysis on *P*. *cynomolgi* and *P*. *inui*, found in high prevalence in the *Anopheles* mosquitoes from this study, had shown close relationship between simian *Plasmodium* from the *Anophele*s mosquitoes with its vertebrate hosts. This directly signifies the ongoing transmission between the vector, macaques, and humans. Furthermore, population genetic analysis showed significant negative values which suggest that both *Plasmodium* species are undergoing population expansion.

**Conclusions/Significance:**

With constant microevolutionary processes, there are potential for both *P*. *inui* and *P*. *cynomolgi* to emerge and spread as a major public health problem, following the similar trend of *P*. *knowlesi*. Therefore, concerted vector studies in other parts of Southeast Asia are warranted to better comprehend the transmission dynamics of this zoonotic simian malaria which eventually would aid in the implementation of effective control measures in a rapidly changing environment.

## Author summary

Increasing knowlesi malaria cases and other zoonotic simian malaria caused by *Plasmodium cynomolgi* and *Plasmodium inui* in humans have added another dimension of complexity to malaria elimination. Unfortunately, the entomological perspective for this disease is scarce and understudied. Accordingly, we aimed to understand the bionomics and transmission efficiency of the simian malaria vectors, and to examine the genetic diversity and evolutionary pattern of their simian *Plasmodium*. Our study revealed that the *Anopheles* Leucosphyrus Group mosquitoes are highly potential competent vectors, and they represent a risk of human infection with zoonotic simian malaria in Southeast Asia. *Plasmodium cynomolgi* and *P*. *inui* were highly prevalent in mosquitoes collected from the present study, and they demonstrated close relationship with those from the vertebrate hosts, suggesting ongoing transmission between the vectors, macaques, and humans. With these constant microevolutionary processes, there are risks for both *P*. *inui* and *P*. *cynomolgi* to emerge and spread as a major public health problem, following the trend of *P*. *knowlesi* in Southeast Asia.

## Introduction

Malaria is one of the most common vector-borne diseases in the world and it is endemic in many countries in tropical and subtropical regions [1]. Southeast Asia is in the pipeline for malaria elimination by 2030 [2]. Increasing cases of zoonotic simian malaria caused by *P*. *knowlesi* pose a new challenge to malaria elimination [3,4]. In addition, cases of natural human infection of other zoonotic simian malaria caused by *P*. *cynomolgi* [5–11] and *P*. *inui* [9,12–14] have added another dimension of complexity to malaria elimination in Southeast Asia. Increasing cases of knowlesi malaria also hinder some of the Southeast Asian countries such as Malaysia from obtaining the malaria-free status certification from the World Health Organization (WHO), though the country had successfully eliminated indigenous human malaria cases since 2018 [15].

One of the contributing factors for the increasing cases of knowlesi malaria and possible emergence of other zoonotic simian malaria is due to extensive deforestation for agricultural activities and expansion of human settlements near forest fringes [16,17]. This potentially brought the spill over of the macaque population to human settlement which eventually caused the mosquito vectors to follow their macaque hosts and adapt to the new environment in semi-urban areas [18]. Unfortunately, the conventional measures to control human-malaria using long-lasting insecticide-impregnated bed nets (LLINs) and indoor residual spraying (IRS) are ineffective in the control of *P*. *knowlesi* transmission [19]. This is especially true since the main vectors for knowlesi malaria are *Anopheles* mosquitoes from the Leucosphyrus Group, which are known to be forest-dwelling mosquitoes [18]. Due to the exophagic and exophilic behaviour of the vectors, outdoor transmission and infective bites just after dusk and in the early morning remain a significant challenge for prevention and control [20–22]. Thus, understanding vector biology is paramount in strategizing effective control measures to combat the rising threat of zoonotic simian malaria. Explicitly, vector distribution, vector competency, adult behaviour and abundance play an important role in determining the transmission potential of the vectors [20].

Despite increasing cases of knowlesi malaria and possible emergence of other zoonotic simian malaria in Southeast Asia, there are limited vector studies to understand the transmission dynamic of this disease. Most of the vector studies have been focused on Malaysian Borneo [18], followed by Vietnam [23,24]. In Peninsular Malaysia, vector studies were conducted in selected locations a decade ago [25,26], and changes in the landscape due to extensive development for the past few decades may have altered the ecology and composition of the *Anopheles* mosquitoes. Thus, there is a dire need for an updated entomological study in this region given the drastic landscape changes due to substantial urbanization. Indeed, the impact of forest disturbance on changes in vector ecology is undeniable and it was well-studied in Malaysian Borneo [16].

In addition, comprehensive entomological studies are also warranted since most of the current vector studies relied heavily on PCR to detect the presence of *Plasmodium* parasites without mosquito dissection. As a result, crucial information such as mosquito parous rate is frequently omitted, which is the key index to estimate mosquito longevity and vector competency [27,28]. Sporozoite rate and entomological inoculation rate were also not deciphered [28]. Thus, there is a major knowledge gap especially on the entomological characteristics of the vectors in Southeast Asia. Furthermore, there is very limited information available regarding the prevalence and genetic diversity of the simian *Plasmodium* isolated from *Anopheles* mosquitoes.

Therefore, with increasing cases of zoonotic simian malaria, it is imperative to conduct a comprehensive entomological study on the local vectors and molecular studies on the simian malaria parasites. Thus, the present longitudinal study was conducted in Malaysia to determine the bionomics and transmission efficiency of the major vectors involved in the transmission of the zoonotic simian malaria parasites, and to evaluate the genetic diversity and evolutionary pattern of the simian *Plasmodium* in them.

## Materials and methods

### Ethics statement

This study was approved by Medical Research and Ethics Committee, Ministry of Health Malaysia (NMRR-19-962-47606). Prior to the study, all volunteers who were involved in the mosquito collections had signed informed consent forms. The participants were asked to immediately report in case they felt ill or developed any symptoms. The project provided free blood examination and treatment of malaria for those who felt ill or wished to check themselves.

### Study area

**Fig 1:**
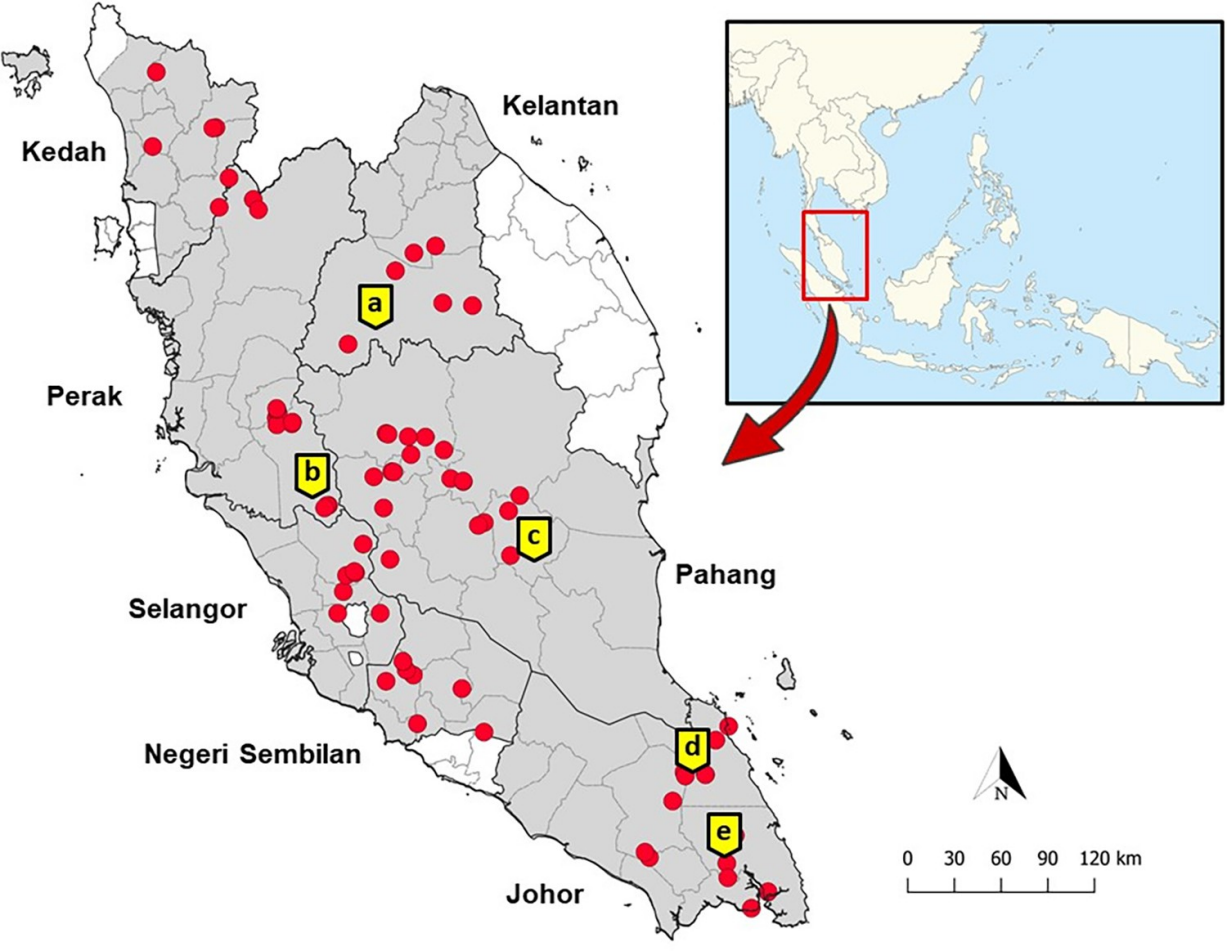
Map of Peninsular Malaysia showing the mosquitoes sampling locations (marked as red dots) with the longitudinal sites for entomological investigations. The longitudinal locations include (a) Kampung Lalang, Kelantan, (b) Sungai Dara, Perak, (c) Kem Sri Gading, Pahang, (d) Bukit Tinggi, Johor and (e) Gunung Panti, Johor. **The map of Peninsular Malaysia was created by the author using QGIS software version 3.6.3 with basemap shapefile modified from the original source on which the data had been plotted (**https://data.humdata.org/dataset/cod-ab-mys**). On the other hand, map of SEA had been sourced out from the public domain, Wikimedia commons (**https://commons.wikimedia.org/wiki/File:Southeast_Asia_location_map.svg).

The study was conducted in seven different states in Peninsular Malaysia between June 2019 and March 2022 (Fig 1). Five longitudinal sampling locations were fixed for entomological investigations to cover a wide geographical range of Peninsular Malaysia. This includes Sungai Dara, Perak (forest fringe) (3°47’46.6"N, 101°31’15.2"E) in northern Peninsular Malaysia and both Kem Sri Gading, Pahang (forest) (3°45’37.9"N, 102°34’20.2"E) and Kampung Lalang, Kelantan (village) (4°54’32.6"N 101°48’58.6"E) in eastern Peninsular Malaysia. On the other hand, in southern Peninsular Malaysia, both Bukit Tinggi (forest) (2°17’14.1"N, 103°40’27.8"E) and Gunung Panti (forest) (1°52’18.4"N 103°52’23.2"E) in Johor were chosen as longitudinal sampling locations. These locations were fixed for entomological investigations after preliminary study showed presence of *Anopheles* mosquitoes from the Leucosphyrus Group which were also positive for the presence of simian malaria parasites. Besides, other random sampling locations were selected based on past human cases of knowlesi malaria and through discussion with the district health officers (S1 Table). In total, there were 81 sampling locations, inclusive of the five longitudinal sampling locations throughout the study.

### Mosquito collection

Adult female *Anopheles* mosquitoes were collected between June 2019 and November 2021 across all the sampling locations using human landing catch (HLC) and Mosquito Magnet as described in previous study [29]. For the longitudinal sampling locations, mosquitoes were collected for two consecutive nights per month from 1900 hours until 2300 hours for four months on a rotational basis between the longitudinal study locations. A total of 8 nights were spent at each longitudinal study location except for Bukit Tinggi, Johor where 10 nights were spent in mosquito collections by a team of two to three trained personnel. All collected *Anopheles* mosquitoes were carefully transported to the field laboratory for identification and were dissected to extract the midgut, salivary glands and ovaries to examine for oocysts, sporozoites and parity status respectively.

### Mosquito identification

All the collected *Anopheles* mosquitoes were morphologically identified to the species level by using the taxonomic keys of Reid [30] and Sallum [31]. For *Anopheles* mosquitoes from the Leucosphyrus Group and mosquitoes which were difficult to be identified morphologically, they were confirmed molecularly through DNA sequencing by amplifying the *ITS2* gene using primers ITS2A and ITS2B [32] with protocol as described in the previous study [33].

### Statistical analysis for entomological indicators

All statistical analysis was carried out using R programming language (version 4.0.0). Parameters such as the abundance of mosquitoes, man-biting rates, parous rate and the proportion of mosquitoes which were infected with sporozoites and oocysts were analyzed using Generalized linear mixed model (GLMM). GLMMs were constructed in R using the glmmTMB package to compare the entomological parameters of *An*. *introlatus* from different sampling locations. In all the analyses, the localities were fitted as a fixed effect while the months of sampling were fitted as random effect. The entomological parameters for *An*. *latens* (n = 23) and *An*. *cracens* (n = 37) were not examined due to overall low density from the sampling locations for robust description.

Poisson distribution was assumed in the analysis of abundance and the man-biting rate of the mosquitoes. On the contrary, binomial distribution was assumed in the analysis of sporozoite rate, oocyst rate and the proportion of parous mosquitoes. Models testing associations between dependent variables (Vector abundance, man-biting rates, parous rates and infection rates) with fixed effect (sampling location) and random effects (months) were compared using higher log-likelihood and lower Akaike information criterion (AIC) values, along with the results of analysis of variance (ANOVA) of nested models. A Tukey’s post-hoc test using multcomp package was used to assess the statistical differences of the entomological parameters between different localities.

### Isolates used for 18S SSU rRNA gene characterisation

All the mosquitoes detected positive for the five simian *Plasmodium* were subjected to nested PCR targeting longer fragments of the 18S SSU rRNA gene for molecular characterisation. Since SSU rRNA gene is a widely used gene marker in eukaryotic phylogeny, there is an abundance of *Plasmodium* species sequence data available [34]. Thus, molecular characterisation of the SSU rRNA gene of the simian *Plasmodium* isolated from mosquitoes will allow comparison of sequence data from both human and macaques to better understand the transmission dynamic of the zoonotic simian malaria in this region. Besides mosquitoes, three macaques infected with simian *Plasmodium* (*P*. *cynomolgi* and *P*. *inui*) from each state were also included in this study. However, in Perak, only one macaque was detected to be positive for *P*. *cynomolgi*. The macaque samples were randomly selected from those captured closest to the mosquito longitudinal sampling locations. All the macaques were collected by the Department of Wildlife and National Parks of Peninsular Malaysia (PERHILITAN) and screened for the presence of simian *Plasmodium* in previous study [35]. In that study, both, *P*. *inui* and *P*. *cynomolgi* were discovered in high prevalence among the macaques in Peninsular Malaysia. The positive samples were then subjected to nested PCR targeting longer fragments in this current study for molecular characterisation together with the DNA sequences of simian *Plasmodium* amplified from mosquitoes.

### Detection of *Plasmodium* parasites in infected mosquitoes and nested PCR assay for *18S SSU rRNA* gene characterisation

The mosquitoes were examined for the presence of sporozoites in the salivary glands and for oocysts in the midgut. Genomic DNA was extracted from the parasite-positive guts and glands using the DNeasy tissue kit (Qiagen, Germany) according to the manufacturer’s protocol. Nested PCR assay was performed targeting the *Plasmodium* small subunit ribosomal RNA (*18S rRNA*) gene to identify human malaria parasites (*Plasmodium falciparum*, *P*. *malariae*, *P*. *ovale curtisi*, *P*. *ovale wallikeri* and *P*. *vivax*) and simian *Plasmodium* (*P*. *coatneyi*, *P*. *cynomolgi*, *P*. *fieldi*, *P*. *inui* and *P*. *knowlesi*) using genus-specific primers rPLU 1 and rPLU 5 for the nest 1 amplification [36], followed by species-specific primers in the nest 2 amplification [37–39] (S2 Table). This protocol has been described in a previous study [29]. The amplification products were analyzed using 1.5% agarose gel electrophoresis.

For gene characterisation targeting the large fragment of the *18S SSU rRNA* gene, the same nest 1 product was used. PCR amplification reaction for nest 2 assay was performed using a universal forward primer UMSF [40] combined with species specific reverse primers [38–40]. PCR amplification reaction for nest 1 assay was performed in a final volume of 50 μL containing 5 μL of DNA template, 1× GoTaq Long PCR Master Mix (Promega, USA), 0.25 μM each of forward (rPLU1) and reverse (rPLU5) primers. Cycling parameter for nest 1 consisted of initial denaturation at 94°C for 4 min, followed by 40 cycles of 94°C for 30s, 55°C for 1 min, 72°C for 1.5 min and a final extension at 72°C for 10 min. For each 25 μL of nest 2 PCR amplification, 3 μL of nest 1 PCR amplification product was used as DNA template. PCR amplification reaction for nest 2 assay consisted of 1× Green GoTaq reaction buffer (Promega, USA), 2.0 mM MgCl_2_ (Promega, USA), 0.2 mM of dNTPs mixture (Promega, USA), 0.4 μM each of forward and reverse primers and 1.5 U of GoTaq DNA polymerase (Promega, USA). Cycling parameter for nest 2 consisted of initial denaturation at 94°C for 5 min, followed by 35 cycles of 94°C for 1 min, species specific annealing temperature for 1 min, 72°C for 1 min and a final extension at 72°C for 5 min (S3 Table).

### Cloning and sequencing of *Plasmodium* 18S SSU rRNA gene fragments

The large fragment of *18S SSU rRNA* gene of the simian *Plasmodium* species isolated from the *Anopheles* mosquitoes and macaques were cloned and sequenced. The amplified PCR product was excised from the agarose gel using a sterile scalpel after gel electrophoresis. The gel slice was purified using NucleoSpin Gel and PCR Clean-up (Macherey-Nagel, Germany) according to the manufacturer’s protocol. Cloning the SSU rRNA gene was done using pEASY-T5 Zero cloning kit (TransGen Biotech, China) according to the manufacturer’s protocol. For transformation, *Trans1*-T1 Phage Resistant Chemically Competent Cell (TransGen Biotech, China) was added to the ligated product. After incubation period, colony PCR was conducted to identify the positive clones with gene insert. Colony PCR was conducted using M13F (F-20) forward primer and M13R (R-26) reverse primer. After the propagation step, the plasmid DNA was extracted using QIAprep Spin Miniprep kit (Qiagen, Germany) according to the manufacturer’s protocol. The extracted plasmids were then sent to 1^st^ Base Laboratories Sdn. Bhd, Malaysia for DNA sequencing.

### Sequence editing, alignment and phylogenetic analysis

The nucleotides sequences of *SSU rRNA* gene of the simian *Plasmodium* parasites obtained from this study were aligned and trimmed using BioEdit version 7.2 software (https://bioedit.software.informer.com/7.2/) while similarity searches were conducted using Basic Local Alignment Search Tool (BLAST) (https://blast.ncbi.nlm.nih.gov/Blast.cgi). Nucleotide sequences of the *SSU rRNA* gene obtained from this study were phylogenetically compared with those obtained from GenBank for the five simian *Plasmodium* species using MEGA version 10.1 software (https://www.megasoftware.net/). Phylogenetic tree was constructed using neighbour-joining method and the evolutionary distances were computed using maximum composite likelihood model with a bootstrap value of 1000 replicates to test the robustness of the tree [41]. In the phylogenetic tree, *Plasmodium berghei* (AJ243513.1) was used as an outgroup.

Nucleotide sequences obtained from cloned samples were aligned with ClustalW using MEGA version 10.1 software (https://www.megasoftware.net/). Sequence analysis and nucleotide comparison were carried out against reference sequence for *P*. *inui* San Antonio I strain (GenBank accession number: XR606809) and *P*. *cynomolgi* Mulligan strain (GenBank accession number: AB287290). For *P*. *inui* and *P*. *cynomolgi*, 171 sequences of PinA-type 18S rRNA (933 bp) and 73 sequences of *PcyA-type 18S rRNA* (927 bp) were respectively used for analysis. The accession number of imported DNA sequences were listed in S4 Table for *P*. *cynomolgi* while S5 Table for *P*. *inui*.

### Haplotype network analysis

The DNA polymorphism of the *A-type 18S SSU rRNA* genes for *P*. *cynomolgi* and *P*. *inui* were estimated by calculating the number of haplotypes (h), haplotype diversity (Hd), nucleotide diversity (π), number of polymorphic sites (k) and average number of pairwise nucleotide differences using DnaSP version 6.12.03 which allows comprehensive analysis of DNA sequence variation. Haplotype networks were also constructed for both *Plasmodium* species based on the *A-type 18S SSU rRNA* partial sequences generated from the present study as well as published sequences from GenBank databases by using the median-joining method in NETWORK version 10.2.0.0 software (Fluxus Technology Ltd, UK). Where available, DNA sequences of reference strains of *P*. *cynomolgi* and *P*. *inui* were included in the construction of the haplotype networks. Details of the published sequences extracted from GenBank databases used in the analysis were listed in S4 and S5 Tables for *P*. *cynomolgi* and *P*. *inui* respectively.

### Population genetic analysis

The 18S SSU rRNA gene was analysed to elucidate the population expansion of simian *Plasmodium* isolated from *Anopheles* mosquitoes. Populations pairwise *F*_ST_ values for genetic distance between the subpopulations were tested for significance using DnaSP version 6.12.03. The value is ranged from 0 to 1 and interpreted as *F*_ST_ = 0 (no differentiation), *F*_ST_ < 0.05 (little differentiation), *F*_ST_ between 0.05 to 0.15 (moderate differentiation), *F*_ST_ between 0.15 to 0.25 (great differentiation) while *F*_ST_ > 0.25 (very great differentiation) [42]. The gene flow was categorized as *N*m < 0.25 (low gene flow), *N*m between 0.25 to 0.99 (intermediate gene flow) while *N*m > 1 (high gene flow) [43]. On the other hand, neutrality test was conducted using Tajima D test [44], Fu and Li D* [45] and Fu and Li F* [46] statistics by using DnaSP version 6.12.03. Under neutrality, Tajima’s D is expected to be 0. Significantly positive Tajima’s D values indicate recent population contraction with selection maintaining variation, whereas negative values suggest population expansion with selection removing variation [44]. Demographic expansion was further investigated with mismatch analysis test using raggedness index (Rag) [47].

## Results

### Abundance of *Anopheles* species

A total of 1652 *Anopheles* mosquitoes belonging to 15 species were caught and examined for the presence of both human and simian *Plasmodium* parasites (Table 1). *Anopheles maculatus* was the predominant species obtained comprising 41.9% of the total catch, followed by *An*. *introlatus* (24.3%), *An*. *letifer* (12.7%) and *An*. *sinensis* (11.1%). Other *Anopheles* species only recorded less than 10% of the total catch. Among the 15 *Anopheles* species obtained throughout the study, *An*. *cracens*, *An*. *introlatus* and *An*. *latens* were the only mosquito species belonging to the Leucosphyrus Group.

**Table 1:** *Anopheles* mosquito species collected from different states in Peninsular Malaysia which were examined for the presence of malaria parasites.

Mosquito species	Johor	Kedah	Kelantan	Negeri Sembilan	Pahang	Perak	Selangor	Total (%)
*An*. *aconitus*	0	6	1	0	9	6	0	22 (1.3)
*An*. *barbirostris* gp.	6	0	0	1	7	4	0	18 (1.1)
*An*. *brevipalpis*	6	0	0	0	0	0	0	6 (0.4)
*An*. *cracens*	0	0	0	0	45	0	0	45 (2.7)
*An*. *epiroticus*	17	0	0	0	0	0	0	17 (1.0)
*An*. *hyrcanus*	2	0	0	0	0	0	0	2 (0.1)
*An*. *introlatus*	367	0	8	7	10	10	0	402 (24.3)
*An*. *karwari*	13	0	0	0	0	0	0	13 (0.8)
*An*. *kochi*	0	2	0	1	0	0	0	3 (0.2)
*An*. *latens*	17	0	10	0	0	0	0	27 (1.6)
*An*. *letifer*	210	0	0	0	0	0	0	210 (12.7)
*An*. *maculatus*	182	22	17	148	28	94	201	692 (41.9)
*An*. *minimus*	0	2	2	1	0	0	0	5 (0.3)
*An*. *sinensis*	0	0	0	0	0	0	183	183 (11.1)
*An*. *tessellatus*	0	0	2	0	5	0	0	7 (0.4)
**Total**	**820**	**32**	**40**	**158**	**104**	**114**	**384**	**1652 (100.0)**

### Species composition of *Anopheles* in all longitudinal study locations

A total of 607 *Anopheles* mosquitoes belonging to 11 different species were collected from five longitudinal study locations across Peninsular Malaysia (Table 2). From those longitudinal sampling locations, *An*. *introlatus* was the predominant species collected which comprised 36.4% of the total catch, followed by *An*. *letifer* and *An*. *maculatus*, each representing 25.9% of the total catch. Other *Anopheles* species were found in lower abundance where each species recorded less than 10% of the total catch.

**Table 2:** *Anopheles* species collected at longitudinal study locations in Peninsular Malaysia.

Mosquito species	Gunung Panti (Forest)	Bukit Tinggi (Forest)	Kg. Lalang (Village)	Kem. Sri Gading (Forest)	Sg. Dara (Forest fringe)	Total (%)
*An*. *aconitus*	0	0	1	0	0	1 (0.2)
*An*. *barbirostris* gp.	0	1	0	4	0	5 (0.8)
*An*. *cracens*	0	0	0	37	0	37 (6.1)
*An*. *epiroticus*	1	0	0	0	0	1 (0.2)
*An*. *hyrcanus*	2	0	0	0	0	2 (0.3)
*An*. *introlatus*	8	189	7	10	7	221 (36.4)
*An*. *latens*	13	0	10	0	0	23 (3.8)
*An*. *letifer*	153	4	0	0	0	157 (25.9)
*An*. *maculatus*	2	74	4	0	77	157 (25.9)
*An*. *minimus*	0	0	1	0	0	1 (0.2)
*An*. *tessellatus*	0	0	2	0	0	2 (0.3)
**Total**	**179**	**268**	**25**	**51**	**84**	**607 (100.0)**

### Seasonal abundance and biting rate of *Anopheles* from longitudinal sites

**Fig 2:**
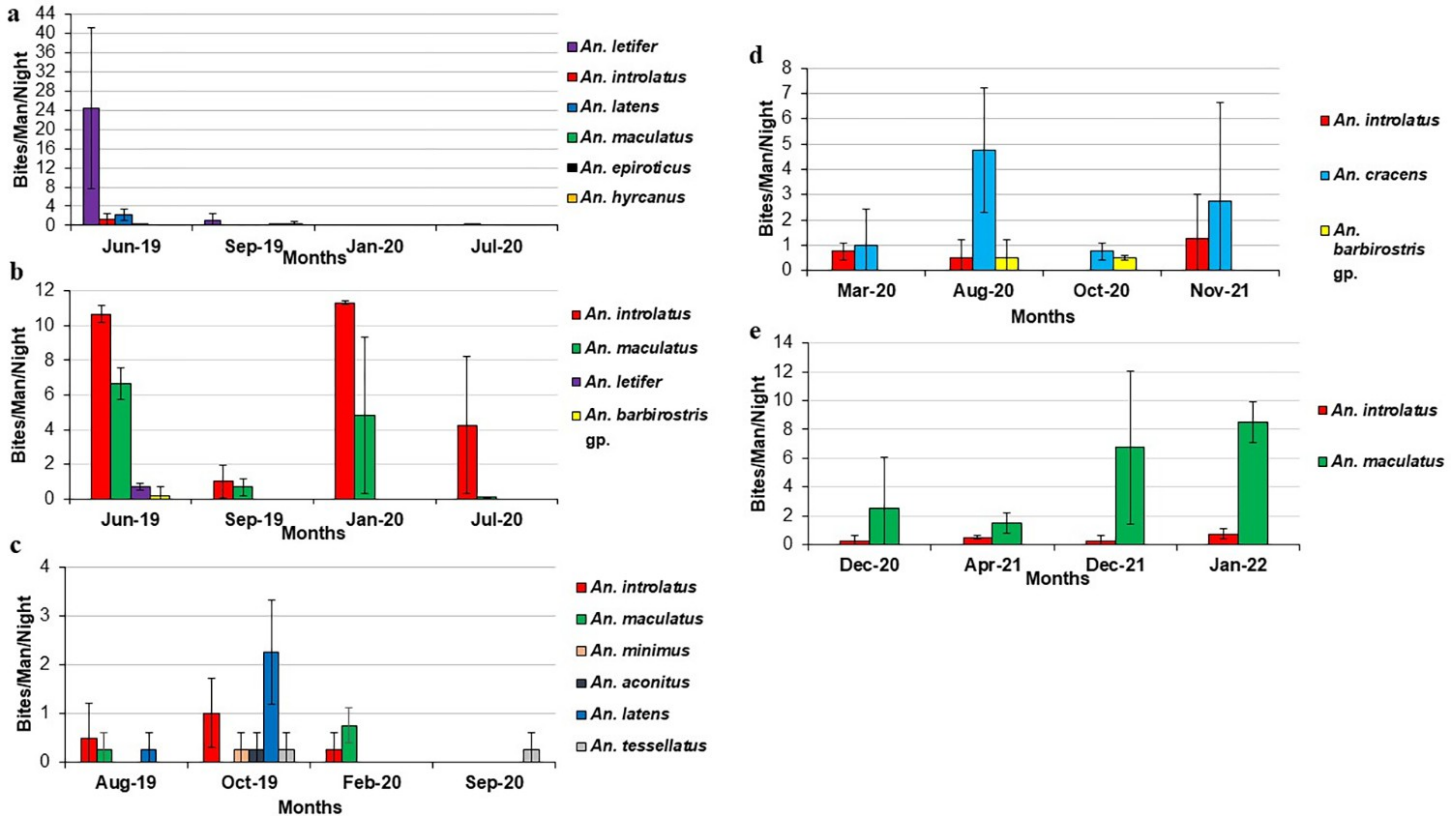
Bites/Man/Night of *Anopheles* mosquitoes at different longitudinal sampling locations. (a) Gunung Panti, Johor, (b) Bukit Tinggi, Johor, (c) Kg. Lalang, Kelantan, (d) Kem Sri Gading, Pahang and (e) Sungai Dara, Perak. **Biting cycles of *Anopheles* from the Leucosphyrus Group.**

The number and species of *Anopheles* mosquitoes vary across different sampling months in each longitudinal sampling location (Fig 2). By using the GLMM models, the results demonstrated that the Poisson distribution was generally a better model than the binomials and negative binomials in analyzing the abundance and biting rate of *An*. *introlatus*. Tukey post hoc test revealed that the abundance and biting rate of *An*. *introlatus* were significantly higher in Bukit Tinggi (forest) compared to other longitudinal sampling locations (P<0.05). By controlling the effect of monthly variation, the mean predicted abundance of *An*. *introlatus* in Bukit Tinggi (forest) was 19 to 28 times higher than other sampling locations, with 12 to 20 times higher mean predicted biting rate.

**Fig 3:**
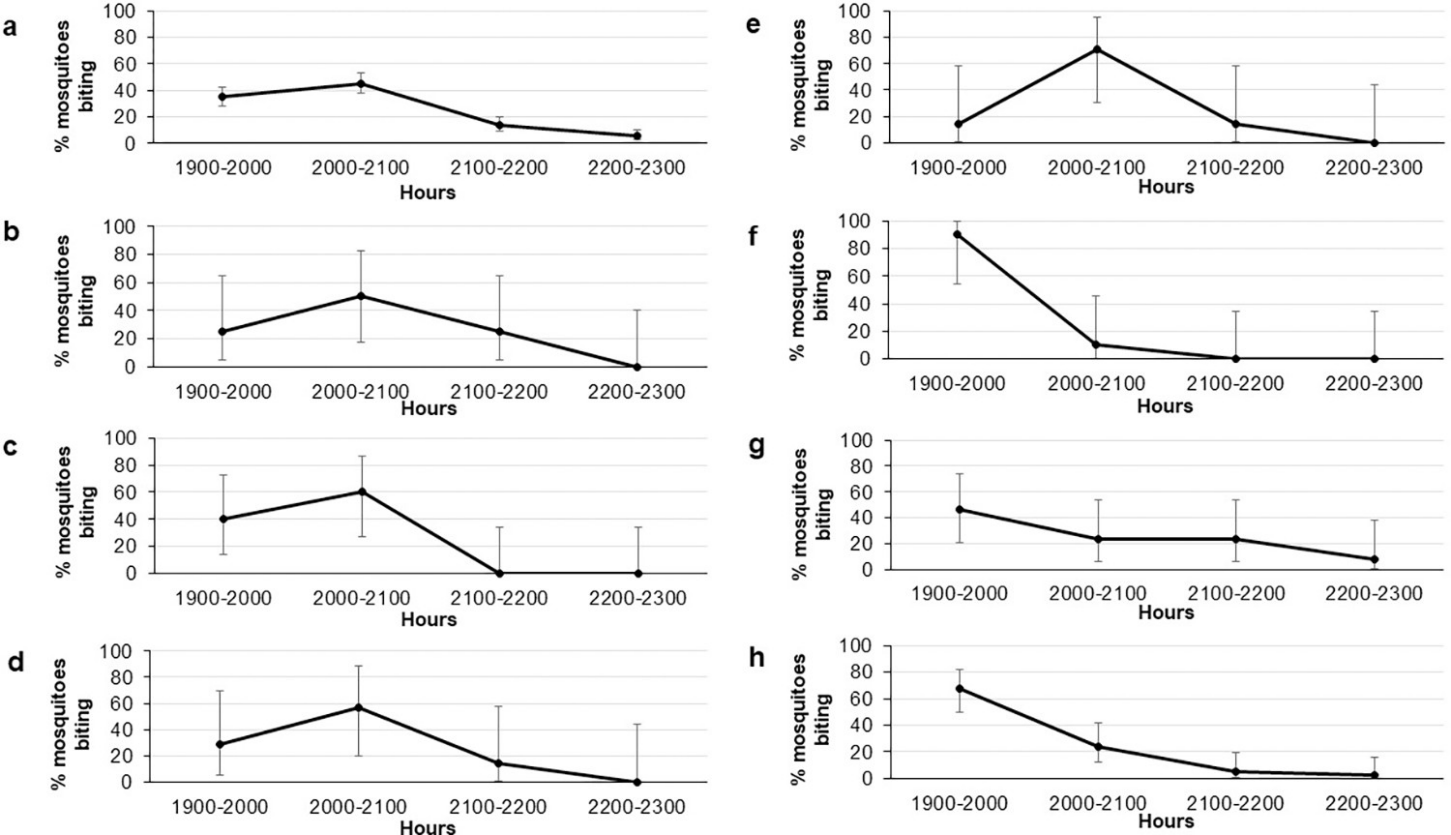
Percentage of *Anopheles* mosquitoes collected hourly at different longitudinal sampling locations. *Anopheles introlatus* collected at (a) Bukit Tinggi, Johor, (b) Gunung Panti, Johor, (c) Kem Sri Gading, Pahang, (d) Kg. Lalang, Kelantan and (e) Sungai Dara, Perak; *Anopheles latens* collected at (f) Kg. Lalang, Kelantan and (g) Gunung Panti, Johor while *An*. *cracens* at (h) Kem Sri Gading, Pahang.

Different peak biting times were observed for the different *Anopheles* species collected from this study (Fig 3). The peak biting time of *An*. *introlatus* was between 2000 to 2100 hours followed by a sharp decline in the percentage of mosquito biting thereafter. Interestingly, the same trend was observed for all *An*. *introlatus* from different longitudinal sampling locations. For *An*. *latens* and *An*. *cracens*, the peak biting time was observed slightly earlier than *An*. *introlatus* which was between 1900 to 2000 hours. This was observed from Kg. Lalang (village) and Gunung Panti (forest) where *An*. *latens* were found and in Kem Sri Gading (forest) for *An*. *cracens*. Generally, all *Anopheles* mosquitoes from the Leucosphyrus Group from this study were early biters with high biting rate between 1900 to 2100 hours with a steady decline thereafter.

### Transmission efficiency of the *Anopheles* Leucosphyrus Group

**Fig 4:**
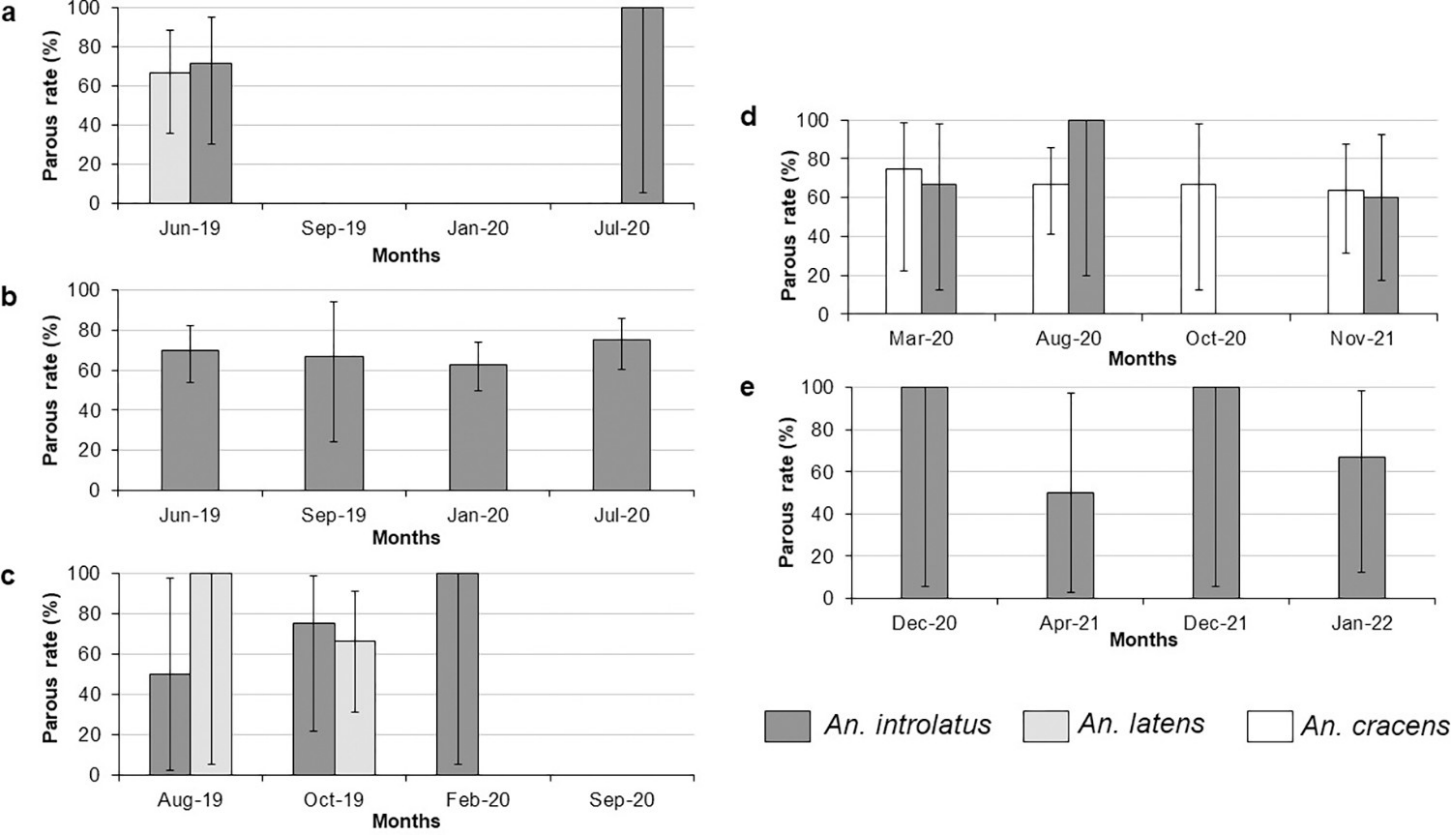
The parous rates of *Anopheles* from the Leucosphyrus Group at longitudinal sampling locations. (a) Gunung Panti, Johor, (b) Bukit Tinggi, Johor, (c) Kg. Lalang, Kelantan, (d) Kem Sri Gading, Pahang and (e) Sungai Dara, Perak.

The parous rates for all three species of *Anopheles* mosquitoes from the Leucosphyrus Group (*An*. *cracens*, *An*. *introlatus* and *An*. *latens*) were predominantly more than 60%. Nonetheless, seasonal variation in the parous rate was not observed for all three species in longitudinal sampling locations (Fig 4). The mean parous rate for *An*. *introlatus* ranged from 68.32% to 75.00% which was slightly higher than *An*. *latens* (66.67% to 70.00%) and *An*. *cracens* (66.67%) (Table 3). Tukey’s test showed no significant difference in the parous rate of *An*. *introlatus* collected from different sampling locations. Statistical test was not employed for the other two species of *Anopheles* since they were only found in some of the longitudinal sampling locations, thus precludes any meaningful comparison.

**Table 3:** The entomological indicators of *Anopheles* from the Leucosphyrus Group from the longitudinal sampling locations.

States	Johor			Kelantan		Pahang		Perak
Sampling locations	Bukit Tinggi	Gunung Panti		Kg. Lalang		Kem. Sri Gading		Sg. Dara
*Anopheles* species	*An*. *introlatus*	*An*. *latens*	*An*. *introlatus*	*An*. *latens*	*An*. *introlatus*	*An*. *cracens*	*An*. *introlatus*	*An*. *introlatus*
**Parameters**								
Number of collected *Anopheles*	189	13	8	10	7	37	10	7
Number of dissected *Anopheles*	161	12	8	10	7	36	10	7
Sporozoite rate (S) (95% CI)	4.23 (1.98–8.47)	–	12.50 (0.66–53.32)	10.00 (0.52–45.88)	–	5.41 (0.94–19.53)	10.00 (0.52–45.88)	14.29 (0.75–57.99)
Ooocyst rate (95% CI)	4.76 (2.34–9.13)	15.38 (2.71–46.34)	–	20.00 (3.54–55.78)	–	–	–	28.57 (5.11–69.74)
Man-biting rate (ma)	6.30	0.54	0.33	0.63	0.44	2.31	0.63	0.44
Entomological inoculation rate (EIR)	0.27	–	0.04	0.06	–	0.12	0.06	0.06
Parity rate (95% CI)	68.32 (60.46–75.30)	66.67 (35.44–88.73)	75.00 (35.58–95.55)	70.00 (35.37–91.90)	71.43 (30.26–94.89)	66.67 (48.95–80.90)	70.00 (35.37–91.90)	71.43 (30.26–94.89)
Probability of daily survival (p)	0.88	0.87	0.91	0.89	0.89	0.87	0.89	0.89
P^10^ (%)	28	26	38	30	33	26	30	33
Life expectancy	7.9	7.4	10.4	8.4	8.9	7.4	8.4	8.9
Infective life	2.2	1.9	4.0	2.5	2.9	1.9	2.5	2.9
Vectorial capacity (VC)	4.60	0.34	0.44	0.53	0.42	1.46	0.53	0.42

Estimation on the probability of daily survival rate, life expectancy and vectorial capacity based on the parous rate were computed for all *Anopheles* mosquitoes from the Leucosphyrus Group at each sampling location (Table 3). Generally, the life expectancy of *An*. *introlatus* was higher compared to *An*. *latens* and *An*. *cracens* from this study. It would be expected that between 28% to 38% of *An*. *introlatus* would survive the 10 days necessary for the formation of *P*. *knowlesi* or other simian *Plasmodium* sporozoites (7.5 days for *P*. *cynomolgi* and 11 days for *P*. *inui*) [48]. Those surviving the 10 days would have a further infective life expectancy of 2.2 to 4.0 days (Table 3). The vectorial capacity was the highest for *An*. *introlatus* from Bukit Tinggi (forest) (4.60) compared to *An*. *introlatus* from other longitudinal sampling locations, mainly influenced by the high man biting rate. The low number of *An*. *introlatus* collected from other longitudinal study locations besides Bukit Tinggi also partially influenced the vectorial capacity of the mosquito.

### Infection rates and entomological inoculation rates by months and sites

**Fig 5:**
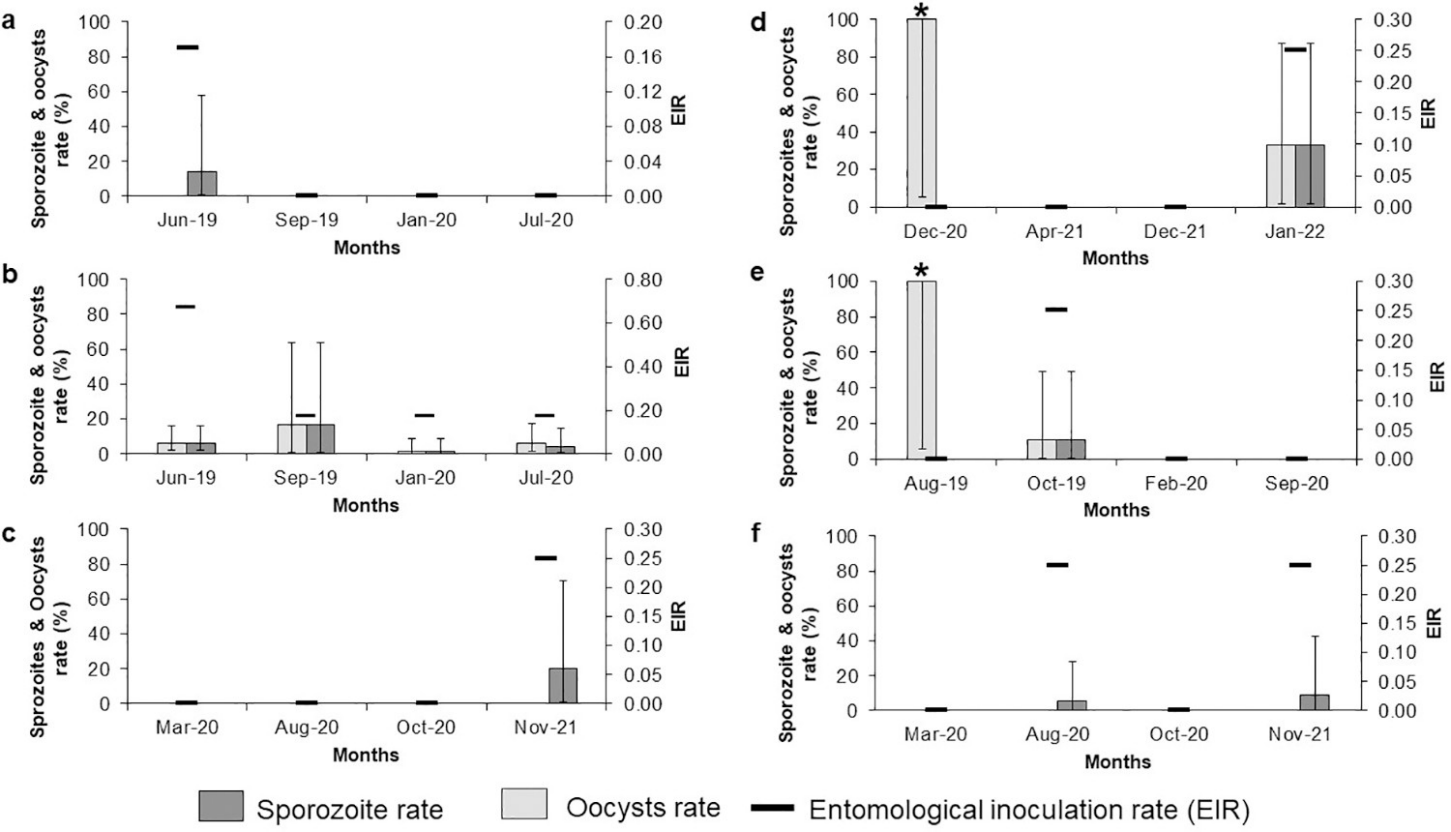
Infection rates and entomological inoculation rates (EIR) of *Anopheles* mosquitoes from the longitudinal sampling locations. *Anopheles introlatus* from (a) Gunung Panti, Johor, (b) Bukit Tinggi, Johor, (c) Kem Sri Gading, Pahang and (d) Sungai Dara, Perak while *An*. *latens* from (e) Kg. Lalang, Kelantan and *An*. *cracens* from (f) Kem Sri Gading, Pahang. *High oocyst rate due to low number of mosquitoes caught.

The oocyst rate and sporozoite rate of the three known simian *Plasmodium* vector species (*An*. *cracens*, *An*. *introlatus* and *An*. *latens*) were assessed. There was no consistent seasonal pattern observed for the infection rates for all the three *Anopheles* species across different study locations (Fig 5). For *An*. *introlatus*, the oocyst rate was extremely high in the month of December 2020 (100%) at Sungai Dara (forest). This was mainly due to low sample size because only a single *An*. *introlatus* was captured and dissected and was found to be positive for the presence of oocysts. A similar scenario was also observed for *An*. *latens* in the month of August 2019 in Kg. Lalang (village) where the oocyst rate was recorded 100%. GLMM analysis with Tukey post hoc tests on *An*. *introlatus* infection rates indicated that there was no significant difference between the infection rate of *An*. *introlatus* among the five different sampling locations. Limited *An*. *latens* (n = 23) and *An*. *cracens* (n = 37) were collected for robust statistical analysis. These two *Anopheles* species were only found in some of the longitudinal sampling locations, thus making it impossible for comparative analysis between different study locations.

Overall, the collected *Anopheles* mosquitoes from the Leucosphyrus Group had an entomological inoculation rate (EIR) between 0.04 to 0.27 (Table 3 and Fig 5). The high EIR was observed in Bukit Tinggi (forest) (0.27) mainly due to high man biting rate. This was followed by *An*. *cracens* at Kem Sri Gading (forest) (0.12) while the lowest EIR was recorded by *An*. *introlatus* from Gunung Panti (forest) (0.04).

### Identification of *Plasmodium* species in mosquito samples

All the collected *Anopheles* species (n = 1652) were examined for the presence of *Plasmodium* parasites through dissection of the mosquitoes to detect the presence of sporozoites or/and oocysts. For those mosquitoes which could not be dissected, the head and thorax were separated from the abdomen, and they were individually screened using PCR to detect the presence of *Plasmodium* parasites. All the positive mosquitoes were mainly from the Leucosphyrus Group except for *An*. *letifer* which was from the Umbrosus Group.

The predominant *Plasmodium* species recovered in this study were *P*. *inui* (n = 33), followed by *P*. *fieldi* (n = 10), *P*. *cynomolgi* (n = 9) and *P*. *coatneyi* (n = 2) (Table 4). For the successfully identified *Plasmodium* species, basic local alignment search tool (BLAST) had 97–100% similarities between all the sequences acquired from this study against the sequences available in GenBank. Most of the positive mosquitoes were of mono-infection (80%), followed by double (18%) and triple infections (2%). The single infections were mainly *P*. *inui* (n = 23). However, the parasite from some mosquito samples, *An*. *letifer* (n = 4), *An*. *introlatus* (n = 2) and *An*. *latens* (n = 1) could only be identified to *Plasmodium* and were negative for human and the five simian malaria parasites species.

**Table 4:** Summary of identified simian *Plasmodium* in *Anopheles* mosquitoes across different states in Peninsular Malaysia.

	Johor		Kelantan		Negeri Sembilan		Pahang		Perak		TOTAL
*Anopheles cracens*	SG	MG	SG	MG	SG	MG	SG	MG	SG	MG	
Pin	NA	NA	NA	NA	NA	NA	2	0	NA	NA	2
											
***Anopheles introlatus***											
*Pct*	2	0	0	0	0	0	0	0	0	0	2
*Pcy*	1	1	0	0	1	0	0	0	0	0	3
*Pfi*	1	2	0	0	0	1	0	0	0	0	4
*Pin*	7	9	0	0	0	0	1	0	1	2	20
*Pcy* + *Pin*	4	1	0	0	0	0	0	0	0	0	5
*Pfi* + *Pin*	1	2	0	0	0	0	0	0	0	0	3
*Plasmodium* sp.	0	2	0	0	0	0	0	0	0	0	2
											
***Anopheles latens***											
*Pin*	0	1	0	0	NA	NA	NA	NA	NA	NA	1
*Pfi*	0	0	1	0	NA	NA	NA	NA	NA	NA	1
*Pin* + *Pfi*	0	0	0	1	NA	NA	NA	NA	NA	NA	1
*Pfi* + *Pcy* + *Pin*	0	0	0	1	NA	NA	NA	NA	NA	NA	1
*Plasmodium* sp.	0	1	0	0	NA	NA	NA	NA	NA	NA	1
											
***Anopheles letifer***											
*Plasmodium* sp.	0	4	NA	NA	NA	NA	NA	NA	NA	NA	4
							
**TOTAL**	16	23	1	2	1	1	3	0	1	2	50

Key: Pct = P. coatneyi, Pcy = P. cynomolgi, Pfi = P. fieldi, Pin = P.inui

Abbreviations: MG = Midgut, SG = Salivary glands, NA = Not available (The species of mosquitoes was not collected/found at the mentioned states)

### Phylogenetic analysis

Phylogenetic tree constructed based on *18S SSU rRNA* gene of the simian *Plasmodium* isolated from *Anopheles* mosquitoes revealed five monophyletic clades with bootstrap values ranging from 59% to 100% (S1 Fig). All the 103 simian *Plasmodium* clones from 54 *Plasmodium* isolates identified from this study were assigned in the phylogenetic trees with their respective species group. The five major clades formed in the phylogenetic tree corresponds to the five simian *Plasmodium* species commonly identified in Malaysia, namely *P*. *coatneyi*, *P*. *cynomolgi*, *P*. *fieldi*, *P*. *inui* and *P*. *knowlesi*. Besides, some of the previously published sequences of simian *Plasmodium* from Malaysian Borneo were also included in the phylogenetic tree (26 sequences). These samples were positioned in the same clade together with samples originating from Peninsular Malaysia based on the species group.

### Haplotype network analysis

**Fig 6:**
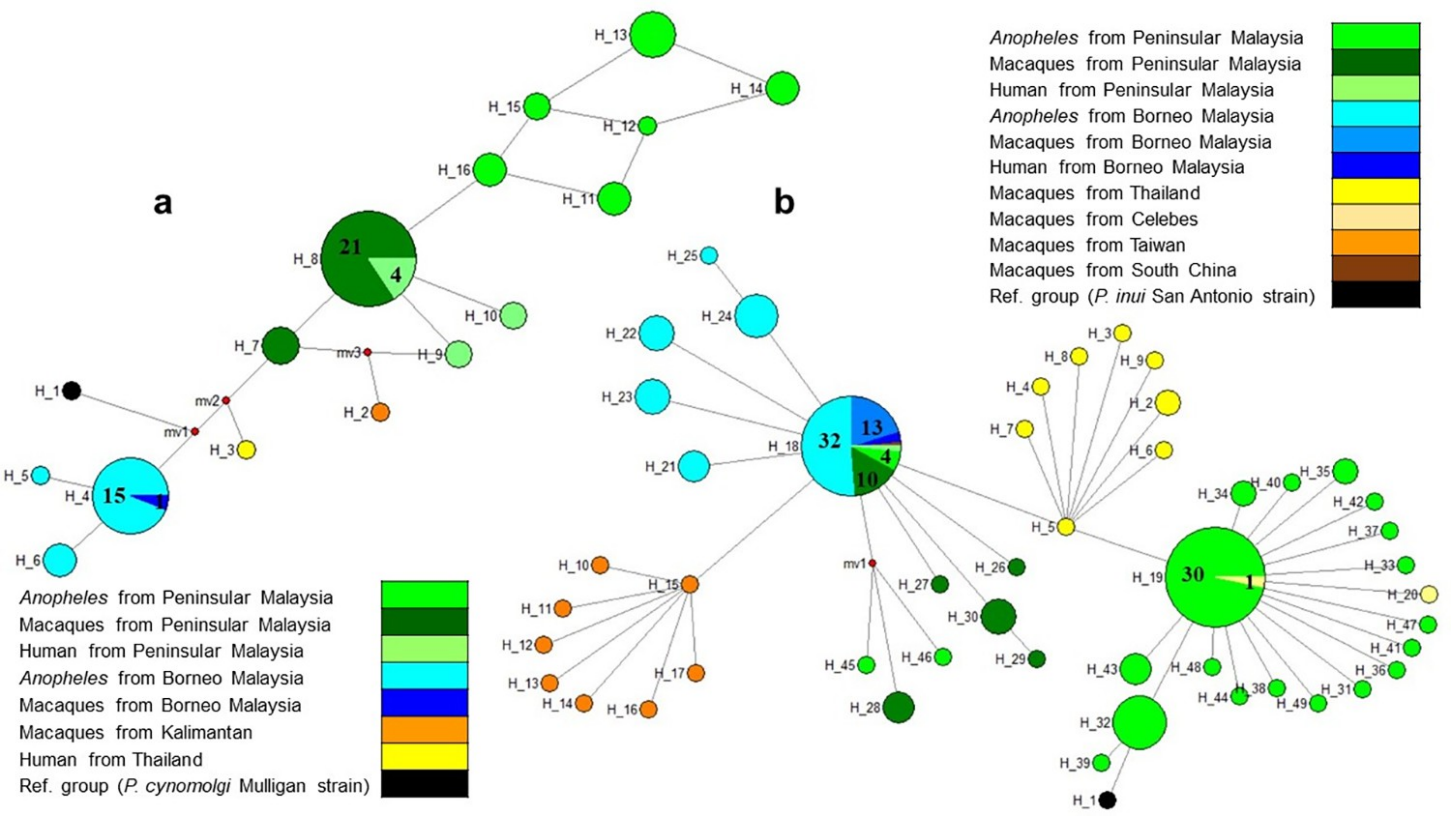
**Median-joining networks of (a) *P*. *cynomolgi* and (b) *P*. *inui* type A small subunit ribosomal *18S RNA* haplotypes.** The network shows relationship among the haplotypes from different hosts in present study (Peninsular Malaysia) as well as other published sequences. The size of the circle representing each haplotype is proportional to the number of sequences that correspond to each respective haplotype while the distances between the nodes are arbitrary.

The haplotype network of *P*. *cynomolgi* (*PcyA-type 18S rRNA*) gene showed geographical clustering between Peninsular Malaysia and Malaysian Borneo which formed two dominant haplotypes, H_8 and H_4 respectively (Fig 6A). The haplotype H_8 was shared between the *P*. *cynomolgi* isolated from macaques and humans from Peninsular Malaysia while haplotype H_4 was shared between macaques and *Anopheles* mosquitoes from Malaysian Borneo. Whereas *P*. *cynomolgi* isolated from *Anopheles* mosquitoes from Peninsular Malaysia (H_11 to H_16) formed their own cluster which is closely related to the dominant haplotypes of macaques and humans from Peninsular Malaysia (H_8). On the other hand, the haplotype network of *P*. *inui* (*PinA-type 18S rRNA*) gene showed two dominant haplotypes (H_18 and H_19) with "star-like" patterns suggesting a population expansion (Fig 6B). Haplotype H_19 mainly consist of *P*. *inui* isolated from *Anopheles* mosquitoes from Peninsular Malaysia. Interestingly, haplotype H_18 was shared by *P*. *inui* isolates from both Peninsular Malaysia and Malaysian Borneo which consisted of samples from all the different hosts (human, macaques, and mosquitoes). Unlike the haplotype network of *P*. *cynomolgi*, there was no clear geographical separation between Peninsular Malaysia and Malaysian Borneo for *P*. *inui* isolates. However, Malaysian samples were clustered separately from the Thailand (H_2 to H_9) and Taiwan (H_10 to H_17) clusters suggesting that the clusters from both neighboring countries were distinctly different populations.

### Population genetic structure

**Fig 7:**
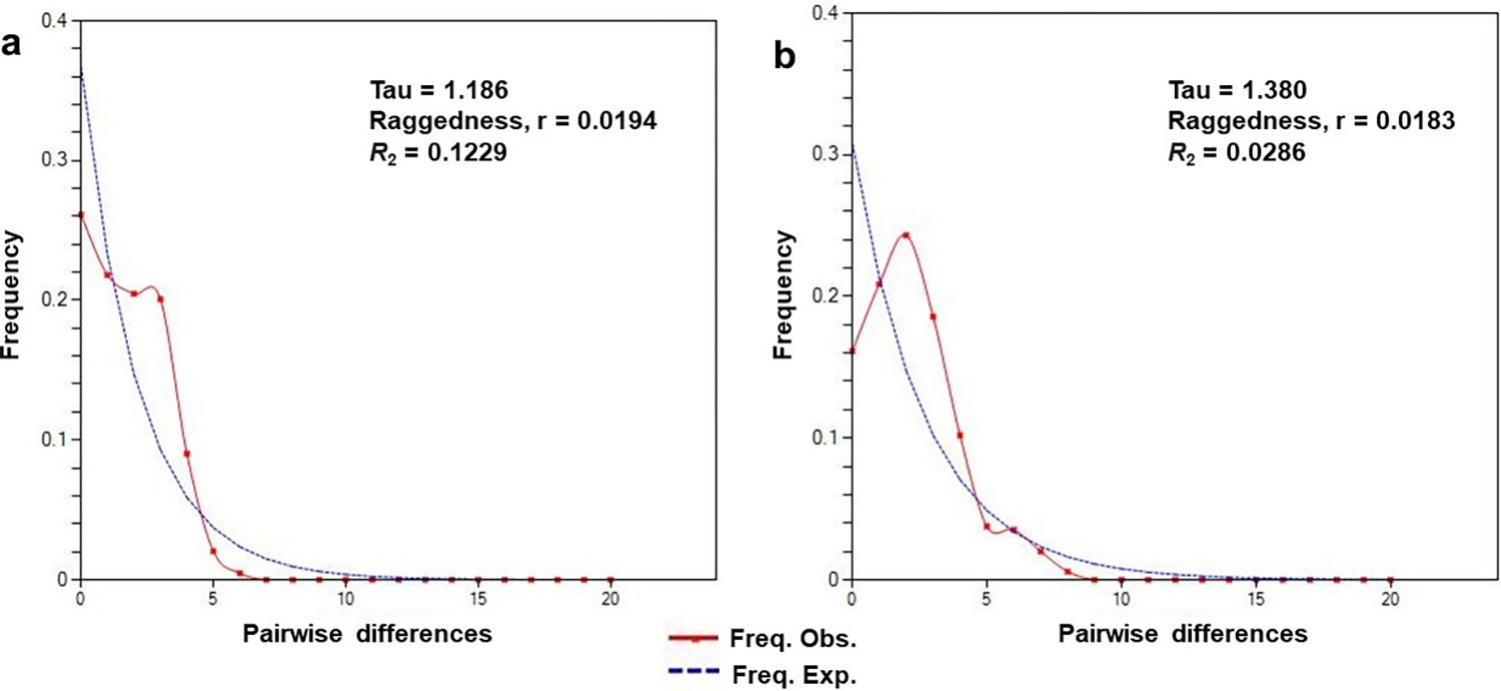
Pairwise mismatch distribution of (a) *P*. *cynomolgi* and (b) *P*. *inui* parasite populations in Peninsular Malaysia based on type A small subunit ribosomal *18S RNA* gene.

Overall, the neutrality tests for both *P*. *cynomolgi* and *P*. *inui* populations showed significant negative values which indicate that the populations are undergoing expansion (S6 Table). In contrast, neutrality tests according to different hosts for *P*. *cynomolgi* isolates from Peninsular Malaysia had shown positive values suggesting recent population contraction due to balancing selection. However, the values were not statistically significant except for Tajima D test for *P*. *cynomolgi* isolated from mosquitoes in Peninsular Malaysia (P < 0.05). For *P*. *inui*, neutrality tests on isolates of *Anopheles* mosquitoes from Peninsular Malaysia showed significant negative values suggesting recent population expansion (P < 0.01). The pairwise distribution analysis for both *P*. *cynomolgi* and *P*. *inui* isolates from Peninsular Malaysia revealed unimodal distribution suggesting population expansion (Fig 7). This was in contrast with the neutrality test which showed positive values for *P*. *cynomolgi* isolates from Peninsular Malaysia irrespective of the different hosts where the parasites had been recovered. However, the values were not statistically significant. On the other hand, the unimodal shape of the pairwise mismatch distribution for *P*. *inui* isolates from Peninsular Malaysia was in parallel with the neutrality test which showed significant negative values suggesting population expansion.

The analysis of pairwise *F*_ST_ values showed significantly high differentiation for the *P*. *cynomolgi* populations isolated from mosquitoes, macaques, and humans in Malaysia. The *P*. *cynomolgi* populations isolated from mosquitoes in Malaysian Borneo showed a very great genetic differentiation as compared to mosquitoes (*F*_ST_ = 0.860), macaques (*F*_ST_ = 0.918) and human (*F*_ST_ = 0.832) from Peninsular Malaysia. The significant genetic differentiation between these two regions of Malaysia suggests the possible geographical isolation that limits the gene flow between the subpopulation of *P*. *cynomolgi* (S7 Table). Similarly, *P*. *inui* populations isolated from mosquitoes in Peninsular Malaysia showed a very great genetic differentiation compared to macaques (*F*_ST_ = 0.683), humans (*F*_ST_ = 0.683) and mosquitoes (*F*_ST_ = 0.601) from Malaysian Borneo. Besides, a high *F*_ST_ values were also obtained when comparing Malaysian populations with populations from other countries such as Thailand (*F*_ST_ > 0.25) and Taiwan (*F*_ST_ > 0.40), high likely due to the geographical distance which limits the gene flow between the subpopulation of *P*. *inui* (S8 Table).

## Discussion

With increasing cases of zoonotic simian malaria in Southeast Asia, it is of paramount importance to identify potential vector species and to understand their bionomics to elucidate human exposure risk to this zoonotic simian malaria. In this study, *An*. *introlatus* was the predominant *Anopheles* species (36.4%) collected from the longitudinal surveillance with mean biting rates ranging from 0.33 to 6.30 bites/man/night. *Anopheles* mosquitoes belonging to the Leucosphyrus Group were the predominant group in each longitudinal sampling location except for Gunung Panti (forest) where the Umbrosus Group was the dominant group. The predominant *Anopheles* species varies across different geographical locations not only in Peninsular Malaysia but also in Southeast Asia. Variation in the species composition and abundance can perhaps be attributed to the environmental heterogeneities [49]. This could also explain why the cases of simian malaria vary widely throughout the region.

A preliminary study conducted in Bukit Tinggi army camp in the forest revealed that none of the *Anopheles* mosquitoes were caught indoors while significantly high number of *Anopheles* mosquitoes were successfully collected outside the camp. This is in parallel with previous studies which showed the exophagic behaviours of *Anopheles* mosquitoes from the Leucosphyrus Group [26,50]. Thus, indoor collection was not carried out throughout the duration of this study. A substantial proportion of *Anopheles* mosquitoes from this study were found biting outdoors in the early evening from 1900–2100 hours, a time when most inhabitants in villages are actively engaged in outdoor activities and unprotected by insecticides treated bed nets [51]. There is also a higher chance of human being exposed to infectious bites when they are returning from daily activities in the forest [52].

Generally, the parous rate for *Anopheles* mosquitoes from this study was high, ranging from 66.67% to 75.00%. The high percentage of parous rate might possibly be due to the suitable climate conditions such as high relative humidity in the area which has a significant impact on the mosquito longevity [27], especially when all the sampling locations were either in forested area or forest fringes with dipterocarp trees forming canopies which provides shade and suitable humid environment for the mosquitoes to thrive. Calculating the parous rate is an important parameter that reflects the age or life expectancy of the mosquito population which has its epidemiological importance in malaria disease transmission [53]. It is only the parous mosquitoes that are likely to transmit malaria because it provides sufficient time for the parasites to develop until the infective sporozoite stage [54].

Although other simian malaria is being reported in small numbers in humans, it should not be dismissed because natural transmission of these zoonotic simian *Plasmodium* is already starting to take root. This includes *P*. *inui* infection in human in Malaysia [13] and *P*. *fieldi* infection in Thailand [12]. While in Kalimantan Indonesia Borneo, human cases of simian malaria caused by *P*. *coatneyi*, *P*. *fieldi* and *P*. *inui* have been postulated [14], which indeed highlight the potential of these simian *Plasmodium* to infect humans in natural settings.

In general, this study revealed that *P*. *inui* was the predominant *Plasmodium* species followed by *P*. *fieldi* and *P*. *cynomolgi* respectively. This observation is in parallel with the prevalence of simian *Plasmodium* recovered from macaques in Malaysia [4]. The over-lapping distribution of the vectors and the macaques in forested areas might explain the high prevalence of these simian *Plasmodium* especially *P*. *cynomolgi* and *P*. *inui* in both the macaques and in the *Anopheles* mosquitoes. Unfortunately, *P*. *knowlesi* was not recovered in any of the *Anopheles* mosquitoes in this current study. Surprisingly, this is also in parallel with few studies in Sabah [55,56] and Sarawak [57] where extremely low number of *P*. *knowlesi* were detected in mosquitoes.

Nevertheless, failure to detect *P*. *knowlesi* in mosquitoes in this study should not be construed as evidence of no transmission. The inability to detect *P*. *knowlesi* in infected *Anopheles* mosquitoes from this study might be due to its low prevalence in mosquitoes as shown in many previous studies [4] or due to selection of sampling locations. Although the sampling locations were selected based on the past knowlesi malaria cases, most of the mosquito samplings were conducted at forest fringes or forested areas accessible by vehicle. However, the infections might have occurred much deeper inside the forest since the villagers who are engaged in forest-related occupation have been reported staying overnight in the deep forest which are usually not accessible by vehicle. Indeed, studies have shown that forest related activities are one of the key risk factors for acquiring *P*. *knowlesi* infections [58]. In addition, a much larger sample size with broader sampling locations covering a range of forest sites may be required to accurately estimate the prevalence of *P*. *knowlesi* in *Anopheles* populations in Peninsular Malaysia. The ability of *Anopheles* vector to transmit other simian *Plasmodium* such as *P*. *cynomolgi* and *P*. *inui* besides *P*. *knowlesi* poses some threat through the emergence of these parasites in future. This is more likely since there were studies highlighting co-infections of these simian malaria parasites with human *Plasmodium* in mosquitoes [23,24,59] indicating possible simultaneous transmission. Since a substantial number of the vectors were positive for *P*. *inui* and *P*. *cynomolgi*, and asymptomatic cases are known to occur [6] it is crucial to screen people in such locations to ensure that they are free of the parasites.

On the other hand, the constructed haplotype network provided insight into the phylogeography clustering of the parasite populations [60]. The shared dominant haplotype of *P*. *inui*, H_18 between mosquito, macaques and human suggests evidence of parasite transmission between the hosts and human. This was further supported by the detection of two natural *P*. *inui* human infections in Pahang, Malaysia together with *P*. *inui* positive *An*. *cracens* mosquito from the same study site [13]. Similar scenario was also observed in the haplotype network of *P*. *cynomolgi* where human and macaques from Peninsular Malaysia shared the same haplotype H_8 while macaques and *Anopheles* from Borneo Malaysia shared another haplotype H_4. Clustering of haplotype of infected *Anopheles* mosquitoes along with the shared haplotypes between different hosts indicate the possible transmission between mosquitoes, humans and macaques in this region. This is especially true when the region had undergone extensive ecological changes due to deforestation giving way for oil palm plantations over the years [61]. Environmental changes especially associated with deforestation and land exploration bring human population in close contact with macaques and forest dwelling *Anopheles* mosquitoes over time, which inevitably increases the risk [62]. This is further supported by recent study on the blood meal of the vectors from the Leucosphyrus Group in this region which exhibit simio-anthropophagic behaviours [63].

Haplotype of *P*. *inui* from Taiwan and Thailand showed distinct geographical clustering away from haplotypes of *P*. *inui* isolated from Peninsular Malaysia and Borneo Malaysia. Indeed, *P*. *inui* isolates from Taiwan [64] and Thailand [65] were collected from a few macaques at sampling sites near each other in their respective countries which eventually leads to the formation of a tight clusters in the haplotype network. Contrarily, the DNA sequences used in the analysis from both Peninsular Malaysia and Borneo Malaysia were collated from the current study and a few different studies conducted many years apart, thus possibly forming a broader cluster [9,66–68]. Besides, the separate clustering of haplotypes derived from macaques from Taiwan could also be due to different species of host where the parasite was isolated. The *P*. *inui* from Taiwan was isolated from Formosan macaques (*Macaca cyclopis*) [64] which was different compared to *P*. *inui* isolated from long-tailed macaques (*Macaca fascicularis*) from other countries. Indeed, a previous study on *P*. *knowlesi* using multilocus microsatellite genotyping had shown two divergent clusters associated with different species of macaque hosts (*M*. *fascicularis* and *M*. *nemestrina*) [69]. Although elucidative and in parallel with previous studies, the conclusion gained from the haplotype analyses remain speculative as they were derived from limited data especially for *P*. *inui* and *P*. *cynomolgi* isolated from humans and macaques. On the other hand, most of the simian *Plasmodium* isolated from vectors in this study originated from southern Peninsular Malaysia which had relatively high sample size of *Anopheles* mosquitoes. Thus, additional studies are required using larger sample size of these zoonotic simian *Plasmodium* species isolated from all the three different hosts from various geographical locations. It is also worth analyzing other polymorphic genetic markers especially from rapidly evolving genes such as *Var* gene [70] or microsatellite loci of the parasites [69] to observe a clear geographical clustering of the parasite populations.

The overall neutrality results for *P*. *cynomolgi* and *P*. *inui* revealed negative values, suggesting both the populations might be undergoing demographic expansion; a similar trend observed for both *P*. *knowlesi* [71] and other human *Plasmodium* species [72,73]. The unimodal mismatch distribution with low values of Raggedness index (P < 0.05) further supported the hypothesis of population expansion for both the simian *Plasmodium* species from this study.

With constant microevolutionary processes, these simian *Plasmodium* will soon be able to adapt and evade the immune responses in wild vector populations and perhaps alter the *Plasmodium* virulence in natural population. Indeed, the effects of genetic variation and changing environmental factors do play a vital role in altering malaria parasite infectivity and *Anopheles* susceptibility to these parasites which eventually affects the emerging infection rates in the future [74]. History has proven that non-human primates do share malaria parasites with humans and host-switching is an ongoing evolutionary process [75]. Thus, if “host-switching” events were to take place, *P*. *knowlesi* and other emerging zoonotic simian malaria caused by *P*. *cynomolgi* and *P*. *inui* might increase in their transmissions and eventually pose a greater threat to the public health in the future. Therefore, besides entomological studies on the vectors, more genetic study on these zoonotic simian *Plasmodium* are warranted to further bolster the understanding on the evolution of these parasites and its interactions between the hosts and vectors which affects its transmission capabilities. Since there is already evidence of simian malarias other than *P*. *knowlesi* being transmitted to humans, proactive measures are needed to prevent escalated risk of infection in the future especially when human malaria is eliminated.

## Supporting information

S1 Fig Phylogenetic tree of *18S SSU rRNA* gene of the positive infected *Anopheles* mosquitoes from the Leucosphyrus Group. Neighbor-joining method was used to construct the phylogeny tree. Number at nodes indicate percentage support of 1000 bootstrap replicates with only bootstrap values above 50% are displayed on the tree. All sequences marked with coloured circles were obtained from this study while sequences marked with coloured triangles were obtained from GenBank.
(DOCX)

S1 Table Sampling locations based on states and districts in Peninsular Malaysia. (DOCX)

S2 Table Oligonucleotide sequence of PCR primers used for detection and identification of *Plasmodium* parasites in mosquitoes. (DOCX)

S3 Table Oligonucleotide sequence of PCR primers used to amplify the 18S SSU rRNA gene of simian malaria parasites in the positive mosquito samples for gene characterisation. (DOCX)

S4 Table Accession numbers of sequences retrieved from GenBank database for *P*. *cynomolgi* which had been included in the analysis. Accession numbers in bold are sequences generated in this study and deposited in GenBank.
(DOCX)

S5 Table Accession numbers of sequences retrieved from GenBank database for *P*. *inui* which had been included in the analysis. Accession numbers in bold are sequences generated in this study and deposited in GenBank.
(DOCX)

S6 Table Neutrality tests on *P*. *cynomolgi* and *P*. *inui* populations isolated from different hosts and locations. (DOCX)

S7 Table Pairwise genetic distance (*F*_ST_) and gene flow (*Nm*) comparisons between subpopulations of *P*. *cynomolgi* parasites based on *18S SSU rRNA* gene. *F*_ST_ values were indicated below the diagonal while the *Nm* values above the diagonals.
(DOCX)

S8 Table Pairwise genetic distance (*F*_ST_) and gene flow (*Nm*) comparisons between subpopulations of *P*. *inui* parasites based on *18S SSU rRNA* gene. *F*_ST_ values were indicated below the diagonal while the *Nm* values above the diagonals.
(DOCX)

## References

[1] WHO. Global malaria report 2020. World Health Organization. 2020.

[2] WHO. Progress towards 0. Malaria-free in South-East Asia region. World Health Organization. 2020.

[3] Zaw MT, Lin Z. Human *Plasmodium knowlesi* infections in South-East Asian countries. J Microbiol Immunol Infect. 2019;52(5):679–84. 31320238 10.1016/j.jmii.2019.05.012

[4] Jeyaprakasam NK, Liew JWK, Low VL, Wan-Sulaiman W-YY, Vythilingam I. *Plasmodium knowlesi* infecting humans in southeast asia: what’s next? PLoS Negl Trop Dis. 2020;14(12):1–16. 10.1371/journal.pntd.0008900 PMC7774830 33382697

[5] Grignard L, Shah S, Chua TH, William T, Drakeley CJ, Fornace KM. Natural human infections with *Plasmodium cynomolgi* and other malaria species in an elimination setting in Sabah, Malaysia. J Infect Dis. 2019;220(12):1946–9. 31418017 10.1093/infdis/jiz397 PMC6834065

[6] Imwong M, Madmanee W, Suwannasin K, Kunasol C, Peto TJ, Tripura R, et al. Asymptomatic natural human infections with the simian malaria parasites *Plasmodium cynomolgi* and *Plasmodium knowlesi*. J Infect Dis. 2019;219(5):695–702. 30295822 10.1093/infdis/jiy519 PMC6376906

[7] Singh B, Kadir KAA, Hu THH, Raja TNN, Mohamad DSS, Lin LWW, et al. Naturally acquired human infections with the simian malaria parasite, *Plasmodium cynomolgi*, in Sarawak, Malaysian Borneo. Int J Infect Dis. 2018;73:68.

[8] Ta TH, Hisam S, Lanza M, Jiram AI, Ismail N, Rubio JM. First case of a naturally acquired human infection with *Plasmodium cynomolgi*. Malar J. 2014;13(1):1–7. 24564912 10.1186/1475-2875-13-68 PMC3937822

[9] Yap NJ, Hossain H, Nada-raja T, Ngui R, Muslim A, Hoh B, et al. Natural human infections with *Plasmodium cynomolgi*, *P*. *inui*, and 4 other simian malaria parasites, Malaysia. Emerg Infect Dis. 2021;27(8):2187–91. 34287122 10.3201/eid2708.204502 PMC8314832

[10] Putaporntip C, Kuamsab N, Pattanawong U, Yanmanee S, Seethamchai S, Jongwutiwes S. *Plasmodium cynomolgi* co-infections among symptomatic malaria patients, Thailand. Emerg Infect Dis. 2021;27(2):590–3. 33496236 10.3201/eid2702.191660 PMC7853550

[11] Sai-ngam P, Pidtana K, Suida P, Poramathikul K, Lertsethtakarn P, Kuntawunginn W, et al. Case series of three malaria patients from Thailand infected with the simian parasite, *Plasmodium cynomolgi*. Malar J. 2022;21(1):1–7. 35524255 10.1186/s12936-022-04167-w PMC9074209

[12] Putaporntip C, Kuamsab N, Seethamchai S, Pattanawong U, Rojrung R, Yanmanee S, et al. Cryptic *Plasmodium inui* and *Plasmodium fieldi* infections among symptomatic malaria patients in Thailand. Clin Infect Dis. 2021;75(5):805–12. 10.1093/cid/ciab1060 34971372

[13] Liew JWK, Bukhari FDM, Jeyaprakasam NK, Phang WK, Vythilingam I, Lau YL. Natural *Plasmodium inui* infections in humans and *Anopheles cracens* mosquito, Malaysia. Emerg Infect Dis. 2021;27(10):2700–3. 34545786 10.3201/eid2710.210412 PMC8462313

[14] Sugiarto SR, Natalia D, Mohamad DSA, Rosli N, Davis WA, Baird JK, et al. A survey of simian *Plasmodium* infections in humans in West Kalimantan, Indonesia. Sci Reports. 2022;12(1):1–11. 10.1038/s41598-022-21570-0 PMC9633791 36329096

[15] WHO. WHO malaria policy advisory group (MPAG) meeting: meeting report, April 2021. 2021.

[16] Fornace KM, Diaz A V, Lines J, Drakeley CJ. Achieving global malaria eradication in changing landscapes. Malar J. 2021;20(1):1–14. 33530995 10.1186/s12936-021-03599-0 PMC7856737

[17] Phang WK, Hamid MHA, Jelip J, Mudin RN, Chuang TW, Lau YL, et al. Spatial and temporal analysis of *Plasmodium knowlesi* infection in Peninsular Malaysia, 2011 to 2018. Int J Environ Res Public Health. 2020;17(24):1–21. 10.3390/ijerph17249271 PMC7764745 33322414

[18] Vythilingam I, Wong ML, Wan-Yussof WS. Current status of *Plasmodium knowlesi* vectors: a public health concern? Parasitology. 2018;145(1):32–40. 27222102 10.1017/S0031182016000901

[19] Scott J. Proposed integrated control of zoonotic *Plasmodium knowlesi* in Southeast Asia using themes of one health. Trop Med Infect Dis. 2020;5(4):175. 33233871 10.3390/tropicalmed5040175 PMC7709578

[20] Suwonkerd W, Ritthison W, Ngo CT, Tainchum K, Bangs MJ, Chareonviriyaphap T. Vector biology and malaria transmission in Southeast Asia. In: *Anopheles* mosquitoes—new insights into malaria vectors. IntechOpen; 2013.

[21] Durnez L, Coosemans M. Residual transmission of malaria: an old issue for new approaches. *Anopheles* mosquitoes—new insights into malaria vectors. IntechOpen; 2013. 671–704.

[22] Fouque F, Knox T. Special programme for research and training in tropical diseases-coordinated multicountry study to determine the burden and causes of residual malaria across different regions. J Infect Dis. 2021; 223:91–8. 23. doi: 10.1093/infdis/jiaa605 33906219 PMC8079130

[23] Marchand RP, Culleton R, Maeno Y, Quang NT, Nakazawa S, Population S, et al. Co-infections of *Plasmodium knowlesi*, *P*. *falciparum*, and *P*. *vivax* among humans and *Anopheles dirus* mosquitoes, Southern Vietnam. Emerg Infect Dis. 2011;17(7):1232–9. 21762577 10.3201/eid1707.101551 PMC3381379

[24] Maeno Y, Quang NT, Culleton R, Kawai S, Masuda G, Nakazawa S, et al. Humans frequently exposed to a range of non-human primate malaria parasite species through the bites of *Anopheles dirus* mosquitoes in South-central Vietnam. Parasit Vectors. 2015;8(1):376. 26178324 10.1186/s13071-015-0995-y PMC4504216

[25] Vythilingam I, Lim YAL, Venugopalan B, Ngui R, Leong CS, Wong ML, et al. *Plasmodium knowlesi* malaria an emerging public health problem in Hulu Selangor, Selangor, Malaysia (2009–2013): epidemiologic and entomologic analysis. Parasit Vectors. 2014;7(1):1–14. 25223878 10.1186/1756-3305-7-436 PMC4261908

[26] Jiram AI, Vythilingam I, Noorazian YM, Yusof YM, Azahari AH, Fong MY. Entomologic investigation of *Plasmodium knowlesi* vectors in Kuala Lipis, Pahang, Malaysia. Malar J. 2012;11(1):1. 22727041 10.1186/1475-2875-11-213 PMC3476358

[27] Adugna T, Getu E, Yewhelew D. Parous rate and longevity of anophelines mosquitoes in bure district, northwestern Ethiopia. PLoS One. 2022;17(2):e0263295. doi: 10.1371/journal.pone.0263295 35120146 PMC8815865

[28] Van de Straat B, Sebayang B, Grigg MJ, Staunton K, Garjito TA, Vythilingam I, et al. Zoonotic malaria transmission and land use change in Southeast Asia: what is known about the vectors. Malar J. 2022;21(1):1–13. 35361218 10.1186/s12936-022-04129-2 PMC8974233

[29] Jeyaprakasam NK, Pramasivan S, Liew JWK, Van Low L, Wan-Sulaiman WY, Ngui R, et al. Evaluation of Mosquito Magnet and other collection tools for *Anopheles* mosquito vectors of simian malaria. Parasit Vectors. 2021;14(1):1–13. 33794965 10.1186/s13071-021-04689-3 PMC8015311

[30] Reid JA. Anopheline mosquitoes of Malaya and Borneo. Stud Inst Med Res Malaysia. 1968;(31):1–520.

[31] Sallum MAM, Peyton EL, Harrison BA, Wilkerson RC. Revision of the Leucosphyrus group of *Anopheles* (Cellia) (Diptera, Culicidae). Rev Bras Entomol. 2005;49(S1):1–152.

[32] Sum JS, Lee WC, Amir A, Braima KA, Jeffery J, Abdul-Aziz NM, et al. Phylogenetic study of six species of *Anopheles* mosquitoes in Peninsular Malaysia based on inter-transcribed spacer region 2 (ITS2) of ribosomal DNA. Parasit Vectors. 2014;7(1):309. 24993022 10.1186/1756-3305-7-309 PMC4094596

[33] Pramasivan S, Ngui R, Jeyaprakasam NK, Wee J, Liew K, Low VL, et al. Spatial distribution of *Plasmodium knowlesi* cases and their vectors in Johor, Malaysia: in light of human malaria elimination. Malar J. 2021;1–12. 34715864 10.1186/s12936-021-03963-0 PMC8555301

[34] Nishimoto Y, Arisue N, Kawai S, Escalante AA, Horii T, Tanabe K, et al. Evolution and phylogeny of the heterogeneous cytosolic SSU rRNA genes in the genus *Plasmodium*. Mol Phylogenet Evol. 2008;47(1):45–53. 18334303 10.1016/j.ympev.2008.01.031

[35] Amir A, Shahari S, Liew JWK, de Silva JR, Khan MB, Lai MY, et al. Natural *Plasmodium* infection in wild macaques of three states in Peninsular Malaysia. Acta Trop. 2020;211:105596. 32589995 10.1016/j.actatropica.2020.105596

[36] Singh B, Bobogare A, Cox-Singh J, Snounou G, Abdullah MS, Rahman HA. A genus- and species-specific nested polymerase chain reaction malaria detection assay for epidemiologic studies. Am J Trop Med Hyg. 1999;60(4):687–92. doi: 10.4269/ajtmh.1999.60.687 10348249

[37] Snounou G, Viriyakosol S, Xin Ping Zhu, Jarra W, Pinheiro L, do Rosario VE, et al. High sensitivity of detection of human malaria parasites by the use of nested polymerase chain reaction. Mol Biochem Parasitol. 1993;61(2):315–20. doi: 10.1016/0166-6851(93)90077-b 8264734

[38] Imwong M, Tanomsing N, Pukrittayakamee S, Day NPJ, White NJ, Snounou G. Spurious amplification of a *Plasmodium vivax* small-subunit RNA gene by use of primers currently used to detect *P*. *knowlesi*. J Clin Microbiol. 2009;47(12):4173–5. 19812279 10.1128/JCM.00811-09 PMC2786678

[39] Lee KS, Divis PCSS, Zakaria SK, Matusop A, Julin RA, Conway DJ, et al. *Plasmodium knowlesi*: reservoir hosts and tracking the emergence in humans and macaques. PLoS Pathog. 2011;7(4):e1002015. 21490952 10.1371/journal.ppat.1002015 PMC3072369

[40] Chua TH, Manin BO, Daim S, Vythilingam I, Drakeley C. Phylogenetic analysis of simian *Plasmodium* spp. infecting *Anopheles balabacensis* Baisas in Sabah, Malaysia. PLoS Negl Trop Dis. 2017;11(10):1–13. 10.1371/journal.pntd.0005991 PMC5638607 28968395

[41] Felsenstein J. Confidence limits on phylogenies: an approach using the bootstrap. evolution. 1985;39(4):783. doi: 10.1111/j.1558-5646.1985.tb00420.x 28561359

[42] Hartl D, Clark A. Principles of population genetics 4th edition (Vol. 116). 4th ed. Sinauer associates; 1997.

[43] Govindaraju DR. Variation in gene flow levels among predominantly self-pollinated plants. J Evol Biol. 1989;2(3):173–81.

[44] Tajima F. Statistical method for testing the neutral mutation hypothesis by DNA polymorphism. Genetics. 1989;123(3):585–95. doi: 10.1093/genetics/123.3.585 2513255 PMC1203831

[45] Fu YX. Statistical tests of neutrality of mutations against population growth, hitchhiking and background selection. Genetics. 1997;147(2):915–25. doi: 10.1093/genetics/147.2.915 9335623 PMC1208208

[46] Fu YX, Li WH. Statistical tests of neutrality of mutations. Genetics. 1993;133(3):693–709. doi: 10.1093/genetics/133.3.693 8454210 PMC1205353

[47] Harpending HC. Signature of ancient population growth in a low-resolution mitochondrial DNA mismatch distribution. Hum Biol. 1994;66(4):591–600. 8088750

[48] Coatney G, Collins WE, Warren W, Contacos PG. The primate malarias. Atlanta Georgia, USA: Centers for Disease Control and Prevention. 1971

[49] Overgaard HJ, Ekbom B, Suwonkerd W, Takagi M. Effect of landscape structure on anopheline mosquito density and diversity in northern Thailand: implications for malaria transmission and control. Landsc Ecol. 2003;18(6):605–19.

[50] Manin BO, Ferguson HM, Vythilingam I, Fornace K, William T, Torr SJ, et al. Investigating the contribution of peri-domestic transmission to risk of zoonotic malaria infection in humans. PLoS Negl Trop Dis. 2016;10(10). doi: 10.1371/journal.pntd.0005064 27741235 PMC5065189

[51] Grigg MJ, Cox J, William T, Jelip J, Fornace KM, Brock PM, et al. Individual-level factors associated with the risk of acquiring human *Plasmodium knowlesi* malaria in Malaysia: a case-control study. Lancet Planet Heal. 2017;1(3):e97–e104. 10.1016/S2542-5196(17)30031-1 PMC5531251 28758162

[52] Wong ML, Chua TH, Leong CS, Khaw LT, Fornace K, Wan-Sulaiman W-YY, et al. Seasonal and spatial dynamics of the primary vector of *Plasmodium knowlesi* within a major transmission focus in Sabah, Malaysia. PLoS Negl Trop Dis. 2015;9(10):e0004135. 26448052 10.1371/journal.pntd.0004135 PMC4598189

[53] Ndoen E, Wild C, Dale P, Sipe N, Dale M. Mosquito longevity, vector capacity, and malaria incidence in West Timor and Central Java, Indonesia. 2012; 2012:1–5.

[54] Williams J, Pinto J. Training manual on malaria entomology for entomology and vector control technicians (basic level) integrated vector management of malaria and other infectious diseases task order 2 contract. 2012;1–78.

[55] Chua TH, Manin BO, Vythilingam I, Fornace K, Drakeley CJ. Effect of different habitat types on abundance and biting times of *Anopheles balabacensis* Baisas (Diptera: Culicidae) in Kudat district of Sabah, Malaysia. Parasit Vectors. 2019;1–13. 31345256 10.1186/s13071-019-3627-0 PMC6659233

[56] Brown R, Salgado-Lynn M, Jumail A, Jalius C, Chua TH, Vythilingam I, et al. Measuring the exposure of primate reservoir hosts to mosquito vectors in Malaysian Borneo. Ecohealth. 2022;1–13. 10.1007/s10393-022-01586-8 PMC9276546 35553290

[57] Tan CH. Identification of vectors of *Plasmodium knowlesi* and other malaria parasites, and studies on their bionomics in Kapit, Sarawak, Malaysia. Master thesis. Universiti Malaysia Sarawak; 2008.

[58] Chin AZ, Avoi R, Atil A, Lukman KA, Rahim SSSA, Ibrahim MY, et al. Risk factor of *Plasmodium knowlesi* infection in Sabah Borneo Malaysia, 2020: a population-based case-control study. PLoS One. 2021;16(9):e0257104. 34506556 10.1371/journal.pone.0257104 PMC8432820

[59] Nakazawa S, Marchand RP, Quang NT, Culleton R, Manh ND, Maeno Y. *Anopheles dirus* co-infection with human and monkey malaria parasites in Vietnam. Int J Parasitol. 2009;39(14):1533–7. 19703460 10.1016/j.ijpara.2009.08.005

[60] Yusof R, Ahmed MA, Jelip J, Ngian HU, Mustakim S, Hussin HM, et al. Phylogeographic evidence for 2 genetically distinct zoonotic *Plasmodium knowlesi* parasites, Malaysia. Emerg Infect Dis. 2016;22(8):1371–80. 27433965 10.3201/eid2208.151885 PMC4982179

[61] Shevade VS, Loboda T V. Oil palm plantations in Peninsular Malaysia: determinants and constraints on expansion. PLoS One. 2019;14(2):e0210628. doi: 10.1371/journal.pone.0210628 30785883 PMC6382120

[62] Fornace KM, Alex, Er N, Abidin TR, Brock PM, Chua TH, et al. Local human movement patterns and land use impact exposure to zoonotic malaria in Malaysian Borneo. Elife. 2019;8(10):1–17. doi: 10.7554/eLife.47602 31638575 PMC6814363

[63] Jeyaprakasam NK, Low VL, Liew JWK, Pramasivan S, Wan-Sulaiman W-Y, Saeung A, et al. Blood meal analysis of *Anopheles* vectors of simian malaria based on laboratory and field studies. Sci Rep. 2022;12(1):1–13. 35013403 10.1038/s41598-021-04106-w PMC8748441

[64] Huang CC, Ji D Der, Chiang YC, Teng HJ, Liu HJ, Chang CD, et al. Prevalence and molecular characterization of *Plasmodium inui* among Formosan macaques (*Macaca cyclopis*) in Taiwan. J Parasitol. 2010;96(1):8–15. 19712012 10.1645/GE-2165.1

[65] Seethamchai S, Putaporntip C, Jongwutiwes S, Cui L, Malaivijitnond S. Malaria and Hepatocystis species in wild macaques, Southern Thailand. Am J Trop Med Hyg. 2008;78(4):646–53. 18385364

[66] Ang JX, Kadir KA, Mohamad DS, Matusop A, Divis PC, Yaman K, et al. New vectors in northern Sarawak, Malaysian Borneo, for the zoonotic malaria parasite, *Plasmodium knowlesi*. Parasit Vectors. 2020;13(1):472. 32933567 10.1186/s13071-020-04345-2 PMC7490903

[67] Divis PC. Identification and molecular characterisation of malaria parasites of macaques in Kapit, Sarawak. Master thesis. Universiti Malaysia Sarawak; 2007.

[68] Wong ML, Ahmed MA, Sulaiman WYW, Manin BO, Leong CS, Quan FS, et al. Genetic diversity of zoonotic malaria parasites from mosquito vector and vertebrate hosts. Infect Genet Evol. 2019;73:26–32. doi: 10.1016/j.meegid.2019.04.010 30999059

[69] Divis PC, Lin LC, Rovie-Ryan JJ, Kadir KA, Anderios F, Hisam S, et al. Three divergent subpopulations of the malaria parasite *Plasmodium knowlesi*. Emerg Infect Dis. 2017;23(4):616. 28322705 10.3201/eid2304.161738 PMC5367419

[70] Reid AJ. Large, rapidly evolving gene families are at the forefront of host-parasite interactions in Apicomplexa. Parasitology. 2015;142(S1) S57–70. doi: 10.1017/S0031182014001528 25257746 PMC4413850

[71] Ahmed MA, Saif A, Quan FS. Diversity pattern of *Plasmodium knowlesi* merozoite surface protein 4 (MSP4) in natural population of Malaysia. PLoS One. 2019;14(11). 10.1371/journal.pone.0224743 PMC6872184 31751362

[72] Han JH, Cho JS, Ong JJY, Park JH, Nyunt MH, Sutanto E, et al. Genetic diversity and neutral selection in *Plasmodium vivax* erythrocyte binding protein correlates with patient antigenicity. PLoS Negl Trop Dis. 2020;14(7):1–16. 10.1371/journal.pntd.0008202 PMC7347095 32645098

[73] Rich SM, Ayala FJ. Population structure and recent evolution of *Plasmodium falciparum*. Proc Natl Acad Sci. 2000;97(13):6994–7001. 10860962 10.1073/pnas.97.13.6994 PMC34375

[74] Tripet F, Aboagye-Antwi F, Hurd H. Ecological immunology of mosquito–malaria interactions. Trends Parasitol. 2008;24(5–3):219. doi: 10.1016/j.pt.2008.02.008 18424235 PMC2474669

[75] Davidson G, Chua TH, Cook A, Speldewinde P, Weinstein P. Defining the ecological and evolutionary drivers of *Plasmodium knowlesi* transmission within a multi-scale framework. Malar J. 2019;18(1):1–13. 30849978 10.1186/s12936-019-2693-2 PMC6408765

