# Supplementary material for: High transmission efficiency of the simian malaria vectors and population expansion of their parasites Plasmodium cynomolgi and Plasmodium inui
Source: PLoS Negl Trop Dis. 2023 Jun 29;17(6):e0011438. doi: 10.1371/journal.pntd.0011438 (PMC10337973; doi:10.1371/journal.pntd.0011438)

**S1 Fig: Phylogenetic tree of *18S SSU rRNA* gene of the positive infected *Anopheles* mosquitoes from the Leucosphyrus Group*.*** Neighbor-joining method was used to construct the phylogeny tree. Number at nodes indicate percentage support of 1000 bootstrap replicates with only bootstrap values above 50% are displayed on the tree. All sequences marked with coloured circles were obtained from this study while sequences marked with coloured triangles were obtained from GenBank.


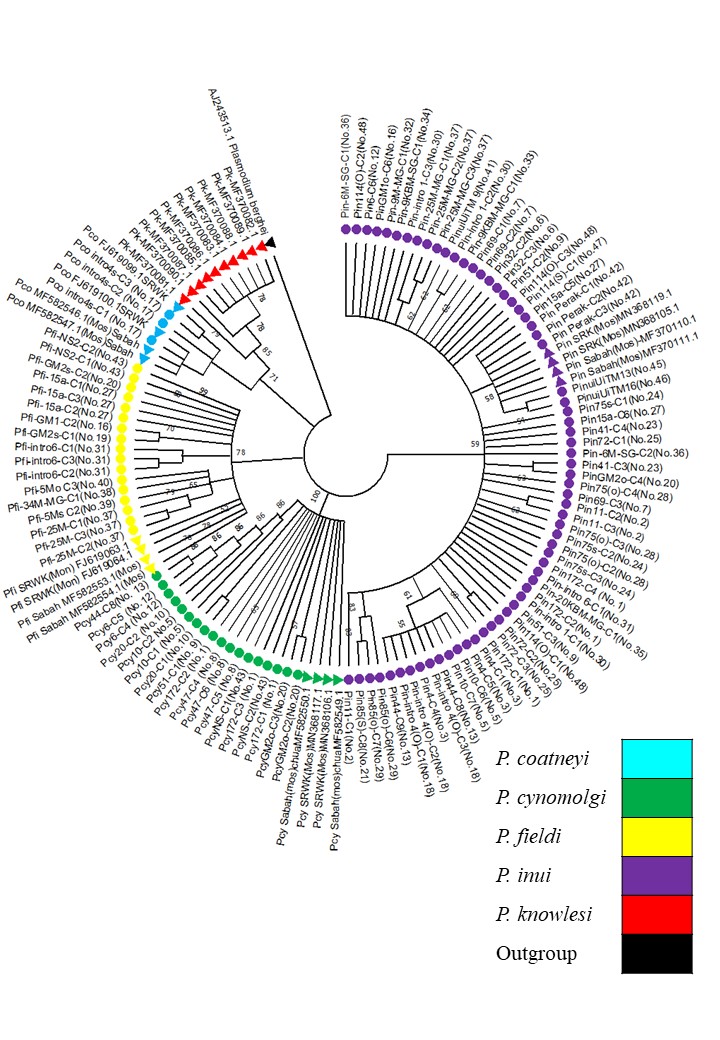

Supplement: S1 Fig — Neighbor-joining method was used to construct the phylogeny tree. Number at nodes indicate percentage support of 1000 bootstrap replicates with only bootstrap values above 50% are displayed on the tree. All sequences marked with coloured circles were obtained from this study while sequences marked with coloured triangles were obtained from GenBank. (DOCX) [file pntd.0011438.s001.docx]
