# Supplementary material for: High transmission efficiency of the simian malaria vectors and population expansion of their parasites Plasmodium cynomolgi and Plasmodium inui
Source: PLoS Negl Trop Dis. 2023 Jun 29;17(6):e0011438. doi: 10.1371/journal.pntd.0011438 (PMC10337973; doi:10.1371/journal.pntd.0011438)
Supplement: S1 Table — (DOCX) [file pntd.0011438.s002.docx]

**S1 Table: Sampling locations based on states and districts in Peninsular Malaysia.**

| **States** | **Districts** | **Sampling locations** | **Latitude** | **Longitude** |
| --- | --- | --- | --- | --- |
| Johor | Kluang | Bukit Nyamuk Simpang Renggam | 1°44’40.2"N | 103°22’41.8"E |
|  |  | Bukit Kenangan | 1°46’31.6"N | 103°21’11.7"E |
|  |  | Kg. Org. Asli Punjut Kahang | 2°14’19.5"N | 103°34’46.6"E |
|  |  | Gunung Belumut | 2°04’18.2"N | 103°30’49.4"E |
|  | Kota Tinggi | Kg. Sri Delima | 1°52’17.4"N | 103°52’21.4"E |
|  |  | Kg. Sri Jaya | 1°42’35.6"N | 103°49’29.3"E |
|  |  | Kg. Sg. Panti | 1°52’17.7"N | 103°52’25.4"E |
|  |  | Kg. Sri Aman | 1°26’53.3"N | 103°57’57.6"E |
|  |  | Kem. Pulada (Army) | 1°37’32.2"N | 103°49’50.9"E |
|  |  | Gunung Panti | 1°52’18.4"N | 103°52’23.2"E |
|  |  | Kg. Org. Asli Bandar Penawar | 1°32’32.9"N | 104°03’40.9"E |
|  |  | Kg. Org. Asli Sg Layau | 1°32’40.1"N | 104°03’43.2"E |
|  | Mersing | Teluk Buih,Pasar Nelayan | 2°30’18.2"N | 103°50’16.6"E |
|  |  | Kongsi balak | 2°13’28.3"N | 103°42’10.8"E |
|  |  | Hutan Lipur Gunung Arong | 2°25’28.3"N | 103°45’48.8"E |
|  |  | Bukit Tinggi Kem Microwave (Army) | 2°17’14.1"N | 103°40’27.8"E |
|  |  | Hutan Lenggor Bukit Tinggi | 2°13’28.3"N | 103°42’10.8"E |
|  |  | Kg. Org. Asli Berasau | 2°13’01.3"N | 103°35’13.0"E |
| Kedah | Baling | Kg. Kaki Bukit | 5°42’16.4"N | 100°57’29.4"E |
|  |  | Kg. Tanjung Luas | 5°32’01.9"N | 100°54’01.2"E |
|  | Padang Terap | Kg. Perik | 6°19’19.7"N | 100°32’13.6"E |
|  | Pendang | Kg. Baloh | 5°53’18.8"N | 100°30’53.9"E |
|  | Sik | Sungai Sok | 5°59’51.0"N | 100°52’58.4"E |
|  |  | Kg. Landai | 5°59’42.1"N | 100°51’53.6"E |
| Kelantan | Gua Musang | Kg. Lalang | 4°54’32.6"N | 101°48’58.6"E |
|  |  | Pos Pasek | 5°09’36.2"N | 101°55’19.2"E |
|  |  | Felda Aring 2 | 4°57’20.7"N | 102°22’03.9"E |
|  |  | Kg. Parik | 4°54’31.8"N | 101°47’19.2"E |
|  |  | Kg. Lebur Jaya | 4°55’14.0"N | 101°48’47.9"E |
|  |  | Chiku 3 | 4°58’17.8"N | 102°11’43.2"E |
|  |  | Kampung Teranek, Bertam | 4°53’23.9"N | 101°47’35.9"E |
| Kelantan | Gua Musang | Kongsi Ladang Sawit Blau | 4°44’05.5"N | 101°38’44.8"E |
|  | Kuala Krai | Kg. Dusun Durian | 5°15’47.8"N | 102°01’46.4"E |
|  |  | Kg. Slow Teming | 5°18’13.3"N | 102°09’19.9"E |
| Negeri sembilan | Jelebu | Hutan Lipur Jeram Toi | 2°48’29.4"N | 102°01’21.7"E |
|  | Kuala Pilah | Ulu Muar | 2°43’43.4"N | 102°17’52.1"E |
|  | Rembau | Kg. Kundur Tengah | 2°31’31.0"N | 102°02’24.2"E |
|  | Seremban | Taman Eko Rimba Lenggeng | 2°50’16.3"N | 101°58’35.1"E |
|  |  | Hutan Lipur Lenggeng | 2°53’14.4"N | 101°57’27.0"E |
|  |  | Hutan Lipur Lenggeng lokasi 2 | 2°53’17.6"N | 101°57’24.9"E |
|  |  | Kebun Durian Tekir | 2°46’21.7"N | 101°51’33.1"E |
|  | Tampin | Kg. Kundur Batang Melaka | 2°28’31.4"N | 102°25’29.2"E |
| Pahang | Bentong | Kongsi Sg. Gabong | 3°28’54.9"N | 101°53’01.9"E |
|  | Jerantut | Kg. Som | 3°57’00.5"N | 102°14’12.7"E |
|  |  | Kg. Lata Kasah | 3°55’58.5"N | 102°18’36.0"E |
|  |  | Hutan Simpan Kenong | 3°56’08.6"N | 102°18’33.1"E |
|  | Kuala Lipis | Kampung Kechau | 4°11’31.7"N | 102°05’30.3"E |
|  |  | Dusun Durian Sg. Koyan | 4°12’53.0"N | 101°51’50.0"E |
|  |  | Kg. Sai | 4°11’41.8"N | 101°59’30.9"E |
|  |  | Kolej Vokasional Kuala Lipis | 4°05’26.0"N | 102°00’26.6"E |
|  |  | Kg. Serunai Mela | 4°07’12.0"N | 102°11’54.0"E |
|  |  | Kg. Sungai Ular | 4°12’35.0"N | 101°52’30.9"E |
|  | Maran | Kem. Sri Gading Jengka | 3°45’37.9"N | 102°34’20.2"E |
|  |  | Kg. Baru Chenor | 3°30’07.7"N | 102°34’52.9"E |
|  |  | Hutan Simpan Berkelah | 3°50’58.3"N | 102°38’12.9"E |
|  | Raub | Kg. Saga | 3°59’28.9"N | 101°53’46.2"E |
|  |  | Kg. Batu Talam | 3°57’43.6"N | 101°47’29.8"E |
|  |  | Taman Rotan Tunggal | 3°46’51.1"N | 101°50’52.8"E |
|  |  | Kg. Segar | 3°59’23.1"N | 101°54’23.1"E |
|  | Temerloh | Gunung Senyum Recreational Forest | 3°41’37.3"N | 102°25’50.1"E |
|  |  | Kuala Krau | 3°40’38.3"N | 102°23’45.1"E |
| Perak | Batang Padang | Tmn. Eden Batu 10 | 4°16’53.3"N | 101°19’17.8"E |
|  |  | Kg. Batu 9 | 4°16’18.2"N | 101°19’03.6"E |
|  |  | Draco Nature Camp, Kg. Sg. Cincin | 4°18’26.9"N | 101°13’39.9"E |
|  |  | Kg. Sungai Genting | 4°20’06.1"N | 101°14’16.5"E |
|  |  | Chenderiang | 4°15’58.2"N | 101°13’53.6"E |
|  | Hulu Perak | Kg. Plang | 5°34’43.9"N | 101°05’55.9"E |
|  |  | Kg. Bongor | 4°16’53.3"N | 101°19’17.8"E |
|  |  | Kg. Kerunai Gerik | 5°31’07.5"N | 101°07’41.2"E |
|  | Kampar | Pos Dipang | 4°21’23.6"N | 101°13’55.0"E |
|  |  | Kg. Sg. Limau | 4°21’42.3"N | 101°13’47.8"E |
|  | Muallim | Kg. Sg. Dara | 3°47’50.6"N | 101°31’44.7"E |
|  |  | Sungai Dara (Near logi air) | 3°47’46.6"N | 101°31’15.2"E |
|  |  | Chinggung 1 | 3°46’56.1"N | 101°30’22.3"E |
| Selangor | Gombak | Commenwealth Forest Park Rawang | 3°17’42.7"N | 101°36’48.6"E |
|  | Hulu Langat | Taman Rekreasi Jeram Tinggi | 3°10’07.3"N | 101°49’34.7"E |
|  | Hulu Selangor | Serendah (Near Chazara Apartment) | 3°23’20.5"N | 101°37’55.5"E |
|  |  | Kg. Org. Asli Pertak | 3°34’15.6"N | 101°43’44.8"E |
|  |  | Sg. Sendat | 3°24’14.8"N | 101°41’03.5"E |
|  |  | Ulu Kalong | 3°24’42.5"N | 101°40’38.5"E |
|  | Petaling | Kota Damansara Community Reserve Forest | 3°10’06.0"N | 101°34’50.7"E |
