# Supplementary material for: High transmission efficiency of the simian malaria vectors and population expansion of their parasites Plasmodium cynomolgi and Plasmodium inui
Source: PLoS Negl Trop Dis. 2023 Jun 29;17(6):e0011438. doi: 10.1371/journal.pntd.0011438 (PMC10337973; doi:10.1371/journal.pntd.0011438)
Supplement: S2 Table — (DOCX) [file pntd.0011438.s003.docx]

**S2 Table: Oligonucleotide sequence of PCR primers used for detection and identification of *Plasmodium* parasites in mosquitoes**

| **Nested PCR** | ***Plasmodium*** | **Primers** | **Sequence (5′ - 3′)** | **Annealing temperature (°C)** | **Expected size (bp)** |
| --- | --- | --- | --- | --- | --- |
| Nest 1 | Genus specific | rPLU 1 | TCAAAGATTAAGCCATGCAAGTGA | 55 | 1640 |
|  |  | rPLU 5 | CCTGTTGTTGCCTTAAACTCC |  |  |
| Nest 2  (Human *Plasmodium*) | *falciparum* | rFAL1 | TTAAACTGGTTTGGGAAAACCAAATATATT | 58 | 205 |
|  |  | rFAL2 | ACACAATGAACTCAATCATGACTACCCGTC |  |  |
|  | *malariae* | rMAL1 | ATAACATAGTTGTACGTTAAGAATAACCGC | 58 | 144 |
|  |  | rMAL2 | AAAATTCCCATGCATAAAAAATTATACAAA |  |  |
|  | *ovale* | rOVA1 | ATCTCTTTTGCTATTTTTTAGTATTGGAGA | 58 | 787 |
|  |  | rOVA2 | GGAAAAGGACACATTAATTGTATCCTAGTG |  |  |
|  | *vivax* | rVIV1 | CGCTTCTAGCTTAATCCACATAACTGATAC | 58 | 117 |
|  |  | rVIV2 | ACTTCCAAGCCGAAGCAAAGAAAGTCCTTA |  |  |
| Nest 2  (Simian *Plasmodium*) | *coatneyi* | PctF1 | CGCTTTTAGCTTAAATCCACATAACAGAC | 60 | 503 |
|  |  | PctR1 | GAGTCCTAACCCCGAAGGGAAAGG |  |  |
|  | *cynomolgi* | CY2F | GATTTGCTAAATTGCGGTCG | 60 | 137 |
|  |  | CY4R | CGGTATGATAAGCCAGGGAAGT |  |  |
|  | *fieldi* | PfldF1 | GGTCTTTTTTTTGCTTCGGTAATTA | 63 | 421 |
|  |  | PfldR2 | AGGCACTGAAGGAAGCAATCTAAGAGTTTC |  |  |
|  | *inui* | PinF2 | CGTATCGACTTTGTGGCATTTTTCTAC | 58 | 479 |
|  |  | INAR3 | GCAATCTAAGAGTTTTAACTCCTC |  |  |
|  | *knowlesi* | Pkf1140 | GATTCATCTATTAAAAATTTGCTTC | 58 | 410 |
|  |  | Pkr1550 | GAGTTCTAATCTCCGGAGAGAAAAGA |  |  |
