# Supplementary material for: High transmission efficiency of the simian malaria vectors and population expansion of their parasites Plasmodium cynomolgi and Plasmodium inui
Source: PLoS Negl Trop Dis. 2023 Jun 29;17(6):e0011438. doi: 10.1371/journal.pntd.0011438 (PMC10337973; doi:10.1371/journal.pntd.0011438)
Supplement: S3 Table — (DOCX) [file pntd.0011438.s004.docx]

**S3 Table: Oligonucleotide sequence of PCR primers used to amplify the 18S SSU rRNA gene of simian malaria parasites in the positive mosquito samples for gene characterisation**

| **Nested PCR** | ***Plasmodium*** | **Primers** | **Sequence (5′ - 3′)** | **Annealing temperature (°C)** | **Expected size (bp)** |
| --- | --- | --- | --- | --- | --- |
| Nest 1 | Genus specific | rPLU 1 | TCAAAGATTAAGCCATGCAAGTGA | 55 | 1640 |
|  |  | rPLU 5 | CCTGTTGTTGCCTTAAACTCC |  |  |
| Nest 2 (Simian *Plasmodium*) | Genus specific (Forward primer) | UMSF^1^ | GGATAACTACGGAAAAGCTGT |  |  |
|  | *P. coatneyi* | PctR1^2^ | GAGTCCTAACCCCGAAGGGAAAGG | 60 | 1029 |
|  | *P. cynomolgi* | CYN1R^1^ | GATTAACTCCGAAGAGAAAATC | 55 | 1015 |
|  | *P. fieldi* | PfldR2^2^ | AGGCACTGAAGGAAGCAATCTAAGAGTTTC | 63 | 1039 |
|  | *P. inui* | INAR3^2^ | GCAATCTAAGAGTTTTAACTCCTC | 60 | 1039 |
|  | *P. knowlesi* | Pkr1550^3^ | GAGTTCTAATCTCCGGAGAGAAAAGA | 50 | 1050 |

References

1. Chua TH, Manin BO, Daim S, Vythilingam I, Drakeley C. Phylogenetic analysis of simian *Plasmodium* spp. infecting *Anopheles balabacensis* Baisas in Sabah, Malaysia. PLoS Negl Trop Dis. 2017;11(10):1–13.
2. Lee KS, Divis PCSS, Zakaria SK, Matusop A, Julin RA, Conway DJ, et al. *Plasmodium knowlesi*: reservoir hosts and tracking the emergence in humans and macaques. PLoS Pathog. 2011;7(4):e1002015.
3. Imwong M, Tanomsing N, Pukrittayakamee S, Day NPJ, White NJ, Snounou G. Spurious amplification of a *Plasmodium vivax* small-subunit RNA gene by use of primers currently used to detect *P. knowlesi*. J Clin Microbiol. 2009;47(12):4173–5.
