# Supplementary material for: High transmission efficiency of the simian malaria vectors and population expansion of their parasites Plasmodium cynomolgi and Plasmodium inui
Source: PLoS Negl Trop Dis. 2023 Jun 29;17(6):e0011438. doi: 10.1371/journal.pntd.0011438 (PMC10337973; doi:10.1371/journal.pntd.0011438)
Supplement: S4 Table — Accession numbers in bold are sequences generated in this study and deposited in GenBank. (DOCX) [file pntd.0011438.s005.docx]

**S4 Table: Accession numbers of sequences retrieved from GenBank database for *P. cynomolgi* which had been included in the analysis.** Accession numbers in bold are sequences generated in this study and deposited in GenBank.

| ***Plasmodium* species** | **Region/ Country** | **Accession number** | | |
| --- | --- | --- | --- | --- |
|  |  | **Mosquito** | **Macaque** | **Human** |
| *P. cynomolgi* | Selangor | NA | MF370174 - MF370183 | NA |
|  | Terengganu | NA | NA | JQ794445 |
|  | Pahang | NA | **ON171441 - ON171444** | NA |
|  | Perak | NA | **ON171437 - ON171440** | MK351409 - MK351413 |
|  | Melaka | NA | NA | MK351416 |
|  | Negeri Sembilan | **ON171703 - ON171704** | NA | MK351414 |
|  | Kelantan | **ON171701 - ON171702** | **ON171430 - ON171433** | NA |
|  | Johor | **ON171687 - ON171700** | **ON171434 - ON171436** | NA |
|  | Sabah | MF370161 - MF370173, MF582549 - MF582552 | NA | NA |
|  | Sarawak | MN368106 - MN368107 | FJ619084 | NA |
|  | Kalimantan | NA | DQ660816 | NA |
|  | Thailand (Denmark traveller) | NA | NA | AB287289 |
