# Supplementary material for: High transmission efficiency of the simian malaria vectors and population expansion of their parasites Plasmodium cynomolgi and Plasmodium inui
Source: PLoS Negl Trop Dis. 2023 Jun 29;17(6):e0011438. doi: 10.1371/journal.pntd.0011438 (PMC10337973; doi:10.1371/journal.pntd.0011438)
Supplement: S5 Table — Accession numbers in bold are sequences generated in this study and deposited in GenBank. (DOCX) [file pntd.0011438.s006.docx]

**S5 Table: Accession numbers of sequences retrieved from GenBank database for *P. inui* which had been included in the analysis.** Accession numbers in bold are sequences generated in this study and deposited in GenBank.

| ***Plasmodium* species** | **Region/ Country** | **Accession number** | | |
| --- | --- | --- | --- | --- |
|  |  | **Mosquito** | **Macaque** | **Human** |
| *P. inui* | Selangor | NA | MF370156 - MF370160 | NA |
|  | Melaka | NA | NA | MK351420 |
|  | Johor | **ON171241 - ON171293** | **ON166785 - ON166788** | NA |
|  | Pahang | **ON171296 - ON171298** | **ON166789 - ON166791** | NA |
|  | Perak | **ON171299 - ON171305** | **ON166792 - ON166794** | NA |
|  | Kelantan | **ON171294 - ON171295** | **ON166795 - ON166799** | NA |
|  | Sabah | MF370110 - MF370155 | NA | NA |
|  | Sarawak | MN368105, MN368114, MN368119, MN368121 | FJ619065, FJ619067, FJ619073, FJ619076, FJ619078 - FJ619079, FJ619081 - FJ619082, FJ619085, FJ619093, FJ619095 - FJ619096, FJ619104 | MK351421 - MK351422 |
|  | Thailand | NA | EU400384 - EU400385, EU400387 - EU400391, EU400395 - EU400396 | NA |
|  | Sulawesi (Celebes) | NA | AB287276 - AB287277 | NA |
|  | Taiwan | NA | FN256224, FN256226 - FN256230, FN430724 - FN430725 | NA |
|  | South China | NA | HM032051 | NA |
