# Supplementary material for: High transmission efficiency of the simian malaria vectors and population expansion of their parasites Plasmodium cynomolgi and Plasmodium inui
Source: PLoS Negl Trop Dis. 2023 Jun 29;17(6):e0011438. doi: 10.1371/journal.pntd.0011438 (PMC10337973; doi:10.1371/journal.pntd.0011438)
Supplement: S6 Table — (DOCX) [file pntd.0011438.s007.docx]

**S6 Table: Neutrality tests on *P. cynomolgi* and *P. inui* populations isolated from different hosts and locations.**

| Plasmodium species | Host | Location | Tajima D | Fu and Li D* | Fu and Li F* |
| --- | --- | --- | --- | --- | --- |
| *P. cynomolgi* | Mosquitoes | Peninsular Malaysia | 2.0061* | 1.02360 | 1.47797 |
|  |  | Malaysian Borneo | -0.77799 | -0.57360 | -0.72018 |
|  | Macaques | Peninsular Malaysia | 0.09498 | 0.61722 | 0.54600 |
|  |  | Malaysian Borneo | NA | NA | NA |
|  |  | Other countries | NA | NA | NA |
|  | Human | Peninsular Malaysia | 0.45766 | 1.23376 | 1.15600 |
|  |  | Malaysian Borneo | NA | NA | NA |
|  |  | Overall total | -0.99031 | -2.87762* | -2.60272* |
| *P. inui* | Mosquitoes | Peninsular Malaysia | -2.38561*** | -4.74428** | -4.62319** |
|  |  | Malaysian Borneo | -0.90105 | 0.13389 | -0.22066 |
|  | Macaques | Peninsular Malaysia | -0.72720 | -0.35425 | -0.53278 |
|  |  | Malaysian Borneo | 0.00000 | 0.00000 | 0.00000 |
|  |  | Other countries | -1.93018* | -2.97351** | -3.10119** |
|  | Human | Peninsular Malaysia | NA | NA | NA |
|  |  | Malaysian Borneo | NA | NA | NA |
|  |  | Overall total | -2.50365**** | -7.99026** | -6.65047** |

Values marked with asterisk indicate significance:
* P < 0.05; **P < 0.02; *** P < 0.01,**** P < 0.001.

NA: Not available - Insufficient DNA sequences to run neutrality test.
