# Supplementary material for: High transmission efficiency of the simian malaria vectors and population expansion of their parasites Plasmodium cynomolgi and Plasmodium inui
Source: PLoS Negl Trop Dis. 2023 Jun 29;17(6):e0011438. doi: 10.1371/journal.pntd.0011438 (PMC10337973; doi:10.1371/journal.pntd.0011438)
Supplement: S7 Table — FST values were indicated below the diagonal while the Nm values above the diagonals. (DOCX) [file pntd.0011438.s008.docx]

**S7 Table: Pairwise genetic distance (*F*_ST_) and gene flow (*Nm*) comparisons between subpopulations of *P. cynomolgi* parasites based on *18S SSU rRNA* gene.** *F*_ST_ values were indicated below the diagonal while the *Nm* values above the diagonals.

| **Location** | **Host** |  | **1** | **2** | **3** | **4** |
| --- | --- | --- | --- | --- | --- | --- |
| Peninsular Malaysia | Mosquitoes | 1 | - | 0.120 | 0.210 | 0.040 |
|  | Macaques | 2 | 0.672*** | - | 1.540 | 0.020 |
|  | Humans | 3 | 0.544** | 0.140** | - | 0.050 |
| Malaysian Borneo | Mosquitoes | 4 | 0.860*** | 0.918*** | 0.832*** | - |

Values marked with asterisk indicate significance: * P < 0.05; ** P < 0.01, *** P < 0.001
