# Supplementary material for: High transmission efficiency of the simian malaria vectors and population expansion of their parasites Plasmodium cynomolgi and Plasmodium inui
Source: PLoS Negl Trop Dis. 2023 Jun 29;17(6):e0011438. doi: 10.1371/journal.pntd.0011438 (PMC10337973; doi:10.1371/journal.pntd.0011438)
Supplement: S8 Table — FST values were indicated below the diagonal while the Nm values above the diagonals. (DOCX) [file pntd.0011438.s009.docx]

**S8 Table: Pairwise genetic distance (*F*_ST_) and gene flow (*Nm*) comparisons between subpopulations of *P. inui* parasites based on *18S SSU rRNA* gene.** *F*_ST_ values were indicated below the diagonal while the *Nm* values above the diagonals.

| **Location** | **Host** |  | **1** | **2** | **3** | **4** | **5** | **6** | **7** | **8** |
| --- | --- | --- | --- | --- | --- | --- | --- | --- | --- | --- |
| Peninsular Malaysia | Mosquitoes | 1 | - | 0.230 | 0.170 | 0.120 | 0.120 | 8.190 | 0.210 | 0.590 |
|  | Macaques | 2 | 0.517*** | - | 2.130 | 1.910 | 1.910 | 0.160 | 0.370 | 0.490 |
| Malaysian Borneo | Mosquitoes | 3 | 0.601*** | 0.105*** | - | 2.980 | 2.980 | 0.100 | 0.320 | 0.400 |
|  | Macaques | 4 | 0.683*** | 0.116 | 0.077 | - | § | 0.060 | 0.280 | 0.330 |
|  | Humans | 5 | 0.683*** | 0.116 | 0.077 | § | - | 0.060 | 0.280 | 0.330 |
| Other countries | Macaques (Celebes) | 6 | 0.030* | 0.605** | 0.705*** | 0.800*** | 0.800 | - | 0.170 | 0.450 |
|  | Macaques (Taiwan) | 7 | 0.548*** | 0.403** | 0.438*** | 0.471** | 0.471 | 0.593 | - | 0.300 |
|  | Macaques (Thailand) | 8 | 0.296*** | 0.340** | 0.384*** | 0.432** | 0.432 | 0.358 | 0.456 | - |

Values marked with asterisk indicate significance: * P < 0.05; ** P < 0.01, *** P < 0.001.

§: No polymorphic sites in the selected region
